# Educational programmes for paediatric healthcare professionals in patient- and family-centred care. A scoping review

**DOI:** 10.1007/s00431-024-05455-0

**Published:** 2024-03-02

**Authors:** Elisabeth Jeppesen, Anne Aarslev Schmidt, Camilla Kriegbaum Skjødt, Jane Hybschmann, Line Klingen Gjærde, Jakob Thestrup, Helena Hansson, Jette Led Sørensen

**Affiliations:** 1 Mary Elizabeth’s Hospital – Rigshospitalet for Children, Teens and Expecting Families, Copenhagen University Hospital – Rigshospitalet, Blegdamsvej 9, 2100 Copenhagen, Denmark; 2 Juliane Marie Centre, Copenhagen University Hospital –Rigshospitalet, Copenhagen, Denmark; 3 Department of Respiratory Medicine, Copenhagen University Hospital - Bispebjerg and Frederiksberg, Copenhagen, Denmark; 4 Department of Pediatric Surgery, Copenhagen University Hospital – Rigshospitalet, Copenhagen, Denmark; 5 Department of Paediatrics and Adolescent Medicine, Copenhagen University Hospital – Rigshospitalet, Copenhagen, Denmark; 6 Department of Clinical Medicine, Faculty of Health and Medical Sciences, University of Copenhagen, Copenhagen, Denmark

**Keywords:** Patient- and family-centred care, Education, Paediatric, Healthcare professionals

## Abstract

To identify and describe educational programmes in patient- and family-centred care for paediatric healthcare professionals. This scoping review was conducted and reported according to the JBI Manual for Evidence Synthesis and the PRISMA guideline. The databases searched included MEDLINE (PubMed), PsycINFO, CINAHL, Scopus, Cochrane, and Embase. Inclusion criteria were experimental, observational and qualitative studies about educational programmes on patient- and family-centred care for paediatric healthcare professionals. Exclusion criteria were reviews and non-peer-reviewed literature. Two reviewers independently screened and extracted the data using Covidence. Of the 13922 records identified, 49 articles met the inclusion criteria. There was a large variety of educational programmes, half of which were interdisciplinary, that mainly targeted nurses and doctors. The median number of participants was 51 (range 7 to 1411). The predominant target population was children with chronic disabilities and neonatal intensive care units, and only one programme specifically targeted adolescents. The median duration was one day (range 5 min to 3.5 years). Development of competencies was the most common objective. We identified 12 different educational content areas. Content mainly focused on communication and relational competencies, including partnership, which involved shared decision-making, mutual agenda setting, and negotiation of a plan. Many kinds of educational strategies were found but experiential learning through simulation and roleplay was used most.

*Conclusion*: A large variety of educational programmes in paediatric patient- and family-centred care exist. Educational content mainly focused on communication and relational competencies. Experiential learning including roleplay and simulation was the most used educational strategy.

**Table:** 

**What is Known:** *• Delivery of patient- and family-centred care improves parental satisfaction of care but requires clinicians have a certain attitude towards involving the child and parents in a healthcare partnership as well as advanced triadic communication skills. Little is known about how this attitude, and more broadly, patient- and family-centred care, can be facilitated through education and training.*	
**What is New:** *• This scoping review found a wide array of programmes.. Workshops with simulation or roleplay was the most frequent educational strategy. The programmes, which typically targeted nurses and doctors, chiefly focused on basic and advanced communication and relational competencies, including partnership, which involved shared decision-making and negotiation of plans.*

**Supplementary Information:**

The online version contains supplementary material available at 10.1007/s00431-024-05455-0.

## Introduction

Patient- and family-centred care is an approach to the planning, delivery, and evaluation of healthcare based on a mutually beneficial partnership among healthcare professionals, patients, and families [1]. Previously known as family-centred care, it has evolved in recent decades, with an increasing focus on children's rights and well-being [1–4]. Various studies report the effect of patient- and family-centred care, and a consistent finding is a higher level of parental satisfaction and shorter hospital stays [5–10]. It has represented a shift from a doctor/nurse-authority approach to shared decision-making with the child and the family. Furthermore, in high income countries it has represented a shift in the culture of care from children being separated from their families when admitted to hospital, to recognising the child and the family as a care unit [2, 3].

A shift in care culture and a sustained behaviour moderation can be facilitated by education [11–13]. The evidence on educational programmes in patient-and family-centred care in paediatrics is limited, with only one narrative review from 2021 available that examines simulation-based learning of patient- and family-centred communication skills within the paediatric setting [14]. However, patient- and family-centred care involves more than communication; it is also a certain attitude toward the patient and family [1]. Hence, the need for a review with a systematic search strategy and a broader scope. The aim of the present scoping review was to identify and describe educational programmes within patient- and family-centred care for paediatric healthcare professionals.

## Materials and methods

This scoping review was conducted according to the JBI Manual for Evidence Synthesis and reported according to the PRISMA guideline for scoping reviews [15, 16]. The protocol was published a priori [17].

We searched MEDLINE (PubMed), PsycINFO, CINAHL, Scopus, Cochrane, and Embase with keywords sorted using the Population/Concept/Context framework. This was done in collaboration with an information specialist [16–18]. Online Resources 1 and 2 in the supplementary material describe the full search strategy and list the keywords. The searches were conducted from 8–11 March 2022 and updated on 4 January 2023. Inclusion criteria were experimental, observational, and qualitative studies published until 2023 evaluating on educational programmes in patient- and family-centred care for healthcare professionals working with paediatric patients 0–18 years of age. There was no time limit or language restrictions. Exclusion criteria were reviews and non-peer-reviewed literature, including conference papers, protocols, and proceedings. Narrative descriptions of educational programmes were not included because we decided only to focus on educational programmes that were evaluated, to get an idea of how they worked.

References were imported to Endnote to remove duplicates before being imported to Covidence. Three reviewers (EJ, AAS, CKS) screened titles/abstracts followed by full text screening. The first author (EJ) screened and read every title/abstract and full text, while AAS and CKS screened and read about half each. When there was a lack of consensus, all three authors met and resolved the conflicts, or involved the last author (JLS). EJ and ASS independently did the charting and consulted JLS when conflicts arose. Conflicts mainly arose when there was uncertainty whether the educational programmes were teaching patient- and family-centred care or not. Critical appraisal of the included studies was not done since it is beyond the aim of a scoping review [16].

Data from eligible studies were charted using a data extraction template developed for this study (Online Resource 3) and included citation, year, country, study design, type and number of healthcare professionals that attended the educational programmes, paediatric population, educational objectives, educational content, educational strategy, duration, if the local need for the education was stated, assessment methods, findings, theoretical framework, accreditation of programmes, and assessed Kirkpatrick level.

We adopted a pragmatic approach on how we included the terms for the concept patient- and family-centred care and included studies with terms belonging to this concept, such as family-centred care and person-centred care [19]. A list of terms can be found in Table 1.

**Table 1:** Characteristics of the 49 included reports

	**Frequency of studies (n)**	**Percentage (%)**
**Publication year** Median = 2018 (1994–2022)		
*< 2000*	2	**4%**
* 2000–2009*	4	**8%**
*2010–2019*	23	**47%**
*2020–2022*	20	**41%**
**Distribution of countries**		
*Australia*	1	**2%**
*Canada*	3	**6%**
*China*	1	**2%**
*Denmark*	4	**8%**
*Finland*	3	**6%**
*Israel*	1	**2%**
*Singapore*	1	**2%**
*South Africa*	1	**2%**
*Switzerland*	1	**2%**
*Turkey*	1	**2%**
*UK*	2	**4%**
*USA*	28	**57%**
*USA/Belgium/Netherlands*	1	**2%**
*USA/Canada*	1	**2%**
**Study design**		
*Pre-post-test*	21	**43%**
*Qualitative*	9	**18%**
*Mixed method*	7	**14%**
*Cross-sectional*	5	**10%**
*Mixed method pre-post-test*	4	**8%**
*Randomised controlled trial*	2	**4%**
*Randomised 2* × *3 experimental design*	1	**2%**
**Theoretical concept**^a^		
*Family-centred care/approach/behaviour/practice/service*	34	**46%**
*Patient/person-centred care/approach/communication*	17	**23%**
*Patient- and family-centred care*	10	**13%**
*Partnerships/patient as partner*	4	**5%**
*Individualised/infant and neuroprotective family-centred developmental care*	3	**4%**
*Care by parent*	1	**1%**
*Child-centred care*	1	**1%**
*Family-integrated care*	1	**1%**
*Guided family-centred care*	1	**1%**
*Patient- and family-centred communication*	1	**1%**
*Relationship-centred communication*	1	**1%**
**Mono- vs interdisciplinary education**		
*Monodisciplinary*	24	**49%**
*Interdisciplinary*	25	**51%**
**Type of healthcare professional**^a^		
*Nurses, nurse practitioners, nursing assistants*	26	**28%**
*Doctors/physicians/surgeons, residents//interns/fellows/house staff*	26	**28%**
*Healthcare students*	14	**15%**
*Occupational therapists*	5	**5%**
*Physiotherapists*	4	**4%**
*Speech therapists*	3	**3%**
*Psychologists*	2	**2%**
*Social workers*	2	**2%**
*Paramedics and emergency medical technicians*	1	**1%**
*Nutritionists*	1	**1%**
*Other*	8	**9%**
**Number of healthcare professionals trained** Median = 51 (7–1411)		
< *30*	10	**20%**
*31*–*50*	13	**27%**
*51*–*100*	8	**16%**
*101*–*200*	7	**14%**
*201*–*400*	5	**10%**
> *401*	3	**6%**
*Not stated*	3	**6%**
**Type of paediatric population**^a^		
*Chronic disabilities/special healthcare needs/rehabilitation*	12	**24%**
* Neonatal intensive care unit*	10	**20%**
*Inpatients*	5	**10%**
*Outpatients*	4	**8%**
*Paediatric manikin/simulator*	4	**8%**
*Infants/toddlers, excluding neonatal intensive care unit*	3	**6%**
*General*	3	**6%**
*Primary care clinic settings*	3	**6%**
*Emergency department*	2	**4%**
*Adolescents*	1	**2%**
*Paediatric intensive care unit*	1	**2%**
*Surgery*	1	**2%**
*Refugees*	1	**2%**
**Single vs multiple sessions**		
*Single*	24	**49%**
*Multiple*	25	**51%**
**Duration of education** Median = 1 day (5 min^b^–3.5 years)		
< *1 h*	2	**4%**
*1–4 h*	14	**29%**
*5–8 h*	2	**4%**
*Half day*	2	**4%**
*One day*	4	**8%**
*2–7 days*	2	**4%**
*About 4 weeks*	5	**10%**
*1–6 months*	6	**12%**
*7–12 months*	2	**4%**
> *1 year*	5	**10%**
*Not stated*	4	**8%**
**Assessment methods**^a^		
*Questionnaires/written evaluation, excluding validated questionnaires*	34	**49%**
*Validated questionnaires*	14	**20%**
*Focus group interviews*	9	**13%**
*Individual interviews*	6	**9%**
*Coding of videotaped interactions/voice recordings/live observations*	7	**10%**
**Kirkpatrick level compiled by research team**^**a,c**^ Median = 2		
*Reaction: 1*	26	**28%**
*Learning: 2*	35	**38%**
*Behaviour: 3*	21	**23%**
*Results: 4*	11	**12%**

^a^The sum is higher than the number of included reports because of several theoretical concepts, paediatric populations, types of healthcare professionals, and assessment methods appear in the same article

^b^5 minutes for neonatal intensive care unit support staff and nursing leaders but part of larger intervention [66]

^c^The Kirkpatrick Model [70] is used to evaluate education and has four levels. Level 1 measures the trainee’s reaction; level 2 knowledge, confidence, or attitude; level 3 behaviour change; and level 4 the effect on an organisational level

## Results

**Fig. 1:**
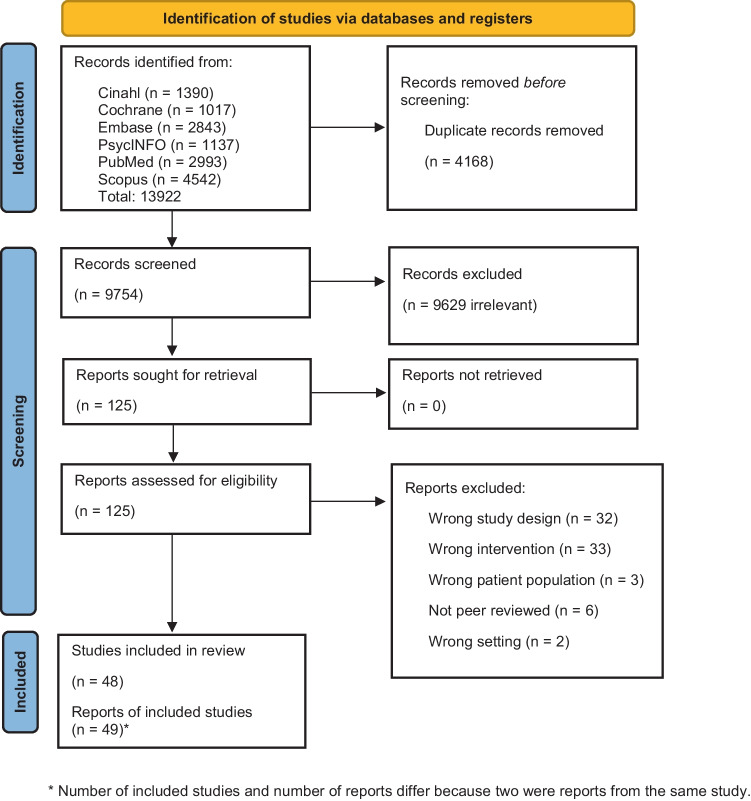
Flowchart

Of the 13922 records we identified, there were 48 eligible studies containing 49 reports (difference due to one study with two reports on the same study (Fig. 1)) [20, 21]. Online Resource 4 provides a full charting of the 49 reports [20–68].

The earliest study included was from 1994 [54]. Half of the studies were published after 2018. Fifteen countries were represented, mainly from high-income countries, with 30 of 49 reports coming from the US. The studies mainly had a pre-post-test design (n = 25), and only two randomised controlled studies were included (Table 1) [23, 67].

A wide variety of terms, related to the term patient- and family-centred care, were used (Table 1). The most applied term was family-centred care. Only 10 of the 49 reports applied the term patient- and family-centred care [26, 28, 30, 44, 47, 49, 52, 56, 60, 68].

About half of the educational programmes (n = 25) were interdisciplinary. Doctors and nurses were primarily the healthcare professionals in the programmes, followed by various types of healthcare students. The median number of healthcare professionals educated was 51 (range 7–1411). The educational programmes mainly involved children with chronic disabilities and the neonatal intensive care unit (NICU), while only one programme primarily targeted adolescents (Table 1).

About half of the educational programmes (n = 25) comprised more than one session. The briefest education lasted five minutes and was for NICU support staff and nursing leaders, though it was part of a larger intervention for the whole staff [66]. The longest educational programme lasted 3.5 years and involved mentoring the staff in the ward [27, 66]. The median duration of the educational programmes was one day (Table 1). Of the 49 reports, 26 (53%) identified the local need for the education.

**Fig. 2:**
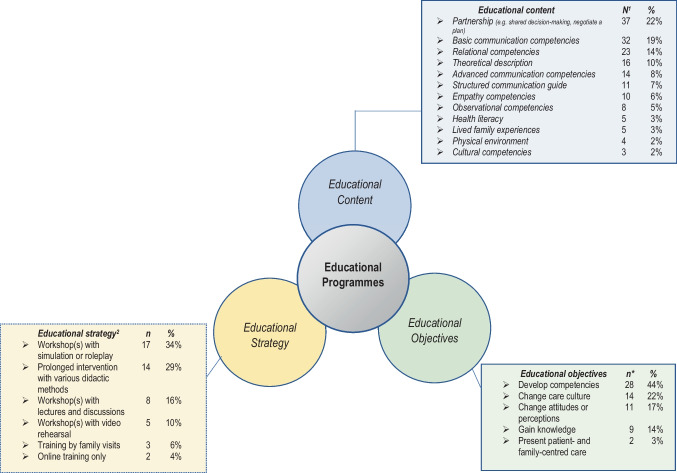
Overview of educational strategy, content, and objectives with frequencies in the 49 included reports interpreted and grouped by the research team. 1) Frequency is higher than number of included studies because several objectives and content areas are in the same programme. 2) For details about the educational strategies see Online Resource 4

We identified and classified five categories of educational objectives in the programmes (Fig. 2). While the most frequent objective was development of competencies within patient- and family-centred care, other objectives were to change care culture, to change attitudes and perceptions, to gain knowledge, and to present patient- and family-centred care.

**Fig. 3:**
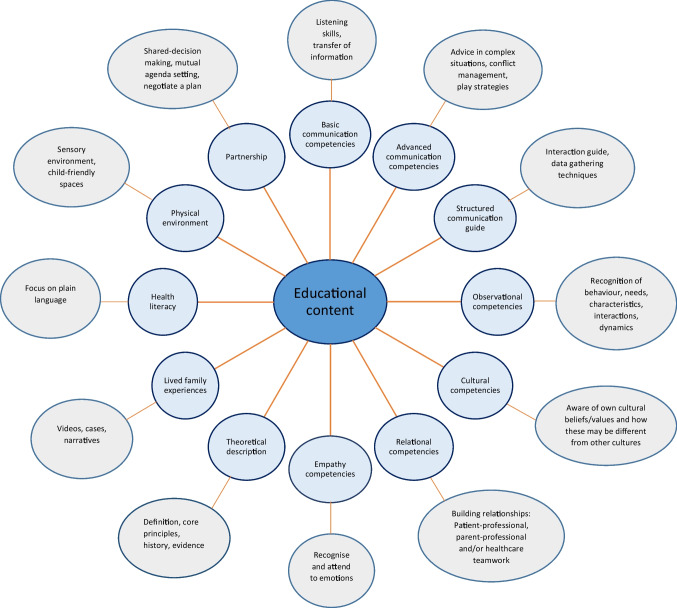
Twelve educational content areas identified in the included educational programmes (light blue circles) with examples (grey outer circles)

We identified and classified 12 educational content areas, which mainly focused on basic and advanced communication and relational competencies (Figs. 2 and 3). Together with communication, the topic most frequently taught was partnership, including shared decision-making and mutual agenda setting (Fig. 2). Topics that received less attention in the educational content were learning about empathy competencies, health literacy, and cultural competencies (Fig. 2). In four of the 49 articles, the educational content covered the physical environment as child-friendly spaces and sensory environments for infants [22, 36, 50, 57].

The most frequent educational strategy was workshops that included simulation or roleplay, sometimes conducted with professional actors (Fig. 2). In addition, prolonged interventions, which included the entire ward and involved various didactic methods, e.g. mentoring and feedback sessions with the staff, were the second most used educational strategy. Some of these programmes lasted over a year [20–22, 27].

Questionnaires and written evaluations were the most frequently used evaluation methods in the studies (Table 1). The participants mainly completed them, but in some studies the parents did, while the children only did so in one study, where the questionnaire did not cover the healthcare professional’s communication skills, but children rated their mental illness symptoms [67]. Fourteen of the studies used various validated questionnaires [26, 33, 37, 38, 45, 46, 50, 51, 54, 56, 60, 63, 67, 68]. Only the Measure of Process of Care for Service Providers questionnaire (MPOC-SP), was used in more than one study [38, 50, 68]. The timing of the evaluation, completed by participants, varied from immediately after the educational programme ended, up to 12 months after the end of the programme. In one study, consultants evaluated the healthcare professionals’ work 12–14 months after the training [22]. Evaluation by parents also varied in timing, with one study counting for the widest range, with a range of 1–1157 days after the end of the educational programme [24]. Further details in Online Resource 4.

We used the Kirkpatrick Model to describe the evaluation of the educational programmes (Tables 1 and 2 and Online Resource 4). The Kirkpatrick Model is often used in medical education to evaluate education and is divided into four levels: reaction, learning, behaviour and results [69].

**Table 2:** Alphabetical list of assessment methods used to evaluate the studies grouped by educational strategy and evaluated using the Kirkpatrick Model

**Educational strategies**	**Assessment methods**	**Frequency**	**Classified by** **Kirkpatrick level** [70]^**a**^			
		(n)	Reaction	Learning	Behaviour	Results
**Training by family visits**	Likert scale (written and oral feedback)	1	X			
	Maternal and Child Health Leadership Competencies (version 3.0)	1	X	X		
	Student reflection paper	1		X		
**Online training only**	Questionnaire	1	X			
	Reflective questions	1		X		
	Videotaped simulated interaction scored by two individual coders and one parent, blinded	1			X	
**Prolonged intervention with various didactic methods**	Activity log	1			X	
	Bliss Baby Charter Audit Tool	1		X	X	X
	California Critical Thinking Disposition Inventory (CCTDI)	1		X		
	Consultation and Relational Empathy Patient Feedback Measure (CARE)	1				X
	Effective Listening and Interactive Communication Scale (ELICS)	1		X		
	Focus group	4	X	X	X	X
	Individual interview	5	X	X	X	
	Jefferson Scale of Patient Perception of Physician Empathy (JSPPPE)	1				X
	Jefferson Scale of Physician Empathy (JSPE)	1		X		
	Kentucky Inventory of Mindfulness Skills (KIMS)	1		X		
	Levenson Locus of Control Scale (Adapted form)	1		X		
	Measure of Processes of Care for Service Providers (MPOC-SP)	1			X	
	Medical errors reviewed by blinded reviewers	1				X
	Mentor review forms	1			X	
	Multidimensional Peer Rating Scale	1			X	
	Peer Nomination Scale of Expertise	1			X	
	Research assistants’ live observations	1			X	
	Rosenberg Self-Esteem Scale	1		X		
	Self-nomination Scale of Expertise in Pediatric Rehabilitation	1			X	
	Questionnaire (not validated)	6	X	X	X	X
	Wee Care assessment survey	1			X	
**Workshop(s) with lectures and discussions**	Focus groups	1	X			
	Measure of Processes of Care - Service Providers (MPOC-SP)	2			X	
	Measure of Processes of Care – Confidence (MPOC-C)	1		X		
	Nurses Attitudes and Behaviors about Rounds questionnaire (NABAR)	1		X	X	
	Questionnaire (not validated)	6	X	X		
**Workshop(s) with simulation or roleplay**	Audiotaped mock counselling sessions analysed with Roter Interaction Analysis System (RIAS)	1			X	
	Focus groups	4		X		
	General Self-Efficacy Scale (GSE)	1		X		
	Hospital Consumer Assessment of Healthcare Providers and Systems (HCAHPS)	1				X
	Individual interview	1				
	Interprofessional Collaborator Assessment Rubric (ICAR)			X		
	Jefferson Scale of Patient Perception of the Health Professionals Empathy (JSPPHPE)	1		X^b^		
	Observation of bedside rounds using checklist (3 observers)	1			X	
	Pediatric Physician Interpersonal Communication Skills Assessment (P-PICSA)	1				X
	Patient-Practitioner Orientation Scale (PPOS)	1				
	Questionnaire (not validated)	12	X	X	X	X
**Workshop(s) with video rehearsal**	Questionnaire (not validated)	4				X
	Strengths and Difficulties Questionnaire^c^	1				X
	Video recorded interview coded by blinded coders	2			X	

^a^The Kirkpatrick Model [70] is used to evaluate education and has four levels. Level 1 measures the trainee’s reaction; level 2 knowledge, confidence, or attitude; level 3 behaviour change; and level 4 the effect on an organisational level

^b^Jefferson Scale of Patient Perception of the Health Professional’s Empathy completed by actors and peers after simulation

^c^Only questionnaire completed by children

Several studies evaluated the education at more than one level (Table 1), but most of them were evaluated on level two (learning) and assessed for whether the programme caused changes in knowledge, skills, attitude, confidence, and commitment (Tables 1 and 2). The least frequent level of evaluation was level 4 (results), which we found in 11 of 49 reports, with intended outcomes described on an organisational level, such as feedback from parents about satisfaction with the communication or quality of care, parent- and youth-rated mental illness symptoms, and functioning or medical errors [21, 23–26, 47, 48, 51, 63, 66, 67]. Table 2 describes which assessment methods we identified within the identified six groups of educational strategies, and which we classified in accordance with Kirkpatrick levels. There was no pattern in how the six groups of educational strategies were evaluated by the four level Kirkpatrick Model (Table 2). The most reported outcome on Kirkpatrick level 4 (results) was parental satisfaction with the communication.

None of the educational programmes were accredited, but one was endorsed by the country’s national health department [57]. Three were certified by a national medical association [23–25].

## Discussion

In summary, this scoping review mainly contains programmes for nurses and doctors that primarily target children with chronic disabilities and the NICU. The most common educational objective was development of competencies within patient- and family-centred care, while the most common educational content was communication competencies and relational competencies, including partnership, which involved shared decision-making and mutual agenda setting. The most used educational strategy was workshops with simulation or roleplay.

A previous narrative review focusing on simulation-based communication training in patient- and family-centred care also found mainly educational programmes designed for nurses and doctors, of various durations, and that addressed various communication challenges [14]. Interestingly, in contrast to this scoping review, they only found few studies that included interprofessional groups and did not evaluate any studies to examine the training on an organisational level (Kirkpatrick level 4).

About half of the studies in this scoping review stated the local need for the education. Need is the first step in the six-step approach for curriculum development [70]. It is crucial for developing effective educational programmes. If need is not assessed, there is a risk that learners will be educated on topics they are already familiar with or skills they have already mastered, or that may not even be applicable. Identifying local needs not only allows to understand the existing local conditions, but also identifies potential areas for modification, how education can be provided, and determines the required components of education. As Johnson et al. concludes there are no “one size fits all” recipe for culture change [13]. As proposed in the six-step approach, we suggest that local needs should always be assessed [70]. Some studies clearly followed the six-step approach for curriculum development for medical education, with one study in particular standing out in terms of adhering to the six-step model by describing each step and distinctly basing the education on local needs [48].

The most common objective was the development of competencies, which offers a concrete objective that is less ambiguous than changing the care culture. A change in the care culture could also be difficult to evaluate when the education takes place separately from the workplace. Moreover, the objective development of competencies seems more feasible to measure in terms of whether goals are achieved than to measure changes in the care culture, which also will be more time consuming. Johnson et al. found in their review of organisational culture change that the majority of the studies focused on knowledge, skills and abilities conducive to change [13].

We outlined 12 areas of educational content, several of which overlap but are defined separately in an attempt to provide an overview of what the various educational programmes taught (Fig. 3). In general, the content of patient- and family-centred care appears to focus on various forms of communication and relationship building. This is in line with the American Academy of Pediatrics Policy Statement of patient- and family-centred care and other articles describing the core values of this type of care [1, 71, 72]. In paediatrics there is a triadic relationship between the patient, family, and healthcare professional. This triadic communication and relationship building requires special communication competencies [73]. Communication is essential in patient- and family-centred care but so is the healthcare professionals’ attitude toward the patient and family [1, 71, 72]. Partnership, which was taught in the majority of the educational programmes (Fig. 2), chiefly focused on the negotiation of a plan, mutual agenda setting, and shared decision-making, which also require special communication competencies and a certain attitude toward the patient and the family. Physical environment understood as child-friendly spaces (Fig. 2) is not traditionally linked to patient- and family-centred care but was included since it was a theme that appeared in four different programmes [22, 36, 50, 57].

The organisational culture is crucial for the quality of the care delivered [13]. About half of the programmes involved interprofessional education, which is suitable in real-life work settings and may enhance the transferability of the programmes [13]. The prolonged interventions involving mentoring and multiple didactic methods in the workplace may also enhance transferability, while short interventions isolated from the clinical setting may be difficult to transfer to the participants’ clinical setting, depending on the opportunities to practice behaviours at work and multiple channels and strategies are likely most effective [13]. The majority of the educational programmes were workshops, possibly because an isolated activity is easier to organise than a prolonged intervention with multiple educational strategies. Despite the likelihood of more success regarding cultural change with a prolonged educational program within the workplace with various didactic methods, there seems to be many ways to culture change and education is just one of the means to achieve culture change [13].

Research on educational programmes in patient- and family-centred care have certain weaknesses in that many of the studies used self-designed questionnaires that were not validated. Only three studies used the same questionnaire, making it difficult to interpret the various studies and to compare the programmes. The most applied study design was a one-group, pre-post-test design with no control group. Only three studies included in this scoping review randomised the participants, and in addition some studies had a low response rate, which could introduce bias. Another weakness is that no children assessed how they experienced their encounter with the healthcare professionals. What the parents experienced may differ from what the child experienced [74]. Most of the studies conducted their evaluation right after the educational programme ended and failed to evaluate whether the education had led to any sustainable changes. As shorter programmes are easier to conduct it seems reasonable that competencies are easier to evaluate than evaluating a sustainable cultural change, which is very complex and requires a lot more effort [13].

Suggestions for future research include evaluating an educational programme within patient- and family-centred care that involves a multicentre, cluster-randomised controlled trial that includes both parents and children responding to the quality of care, blinded to the intervention, and where the outcome is measured on a validated instrument with long-term follow-up. In addition, a detailed description of content in the educational programme. This is lacking in some of the included studies, which makes it difficult to interpret what the content exactly is. A contribution in future studies can be to apply frameworks and standards for development of educational programmes [70].

Implications for practice involve finding inspiration in the educational programmes included in this scoping review. However, it is essential to always align with local needs [13, 70]. When developing new programmes, there is a scarcity of initiatives designed for teenagers, other healthcare professionals besides doctors and nurses, and prolonged interventions aiming to change the care culture.

This scoping review has several strengths and limitations. One of the strengths is that the review follows the scoping review framework regarding a systematic search and selection [15, 16]. In addition, the study protocol was published a priori [17]. Six major databases were searched, and the search strategy was designed in collaboration with an information specialist. The selection process was pragmatic, took an inclusive approach regarding the concept of patient- and family-centred care, and had no language restriction or time limit, leading to the creation of a rich material.

There are, however, also limitations to consider. For example, the pragmatic approach taken in the selection process regarding the concept of patient- and family-centred care can also be seen as a limitation, including too broad a range of educational programmes. This may lead to uncertainty concerning the concept’s core attributes, making the concept diffuse, which may hamper compilation and comparison. Some may argue that each concept is unique and cannot be combined or compared, but the authors of this study assert that taking a pragmatic approach is necessary to gain an overview and promote transferability to various clinical settings, as the terms share core attributes. It would be a scoping review with just 10 studies if we limited the concept to only include “patient- and family-centred care” straightforward. So taken together the authors of this review views the pragmatic choice more as a strength than a limitation.

The inclusion criteria required that the studies had to have an experimental, observational or qualitative design concerning the evaluation of the programme, which may have led to the exclusion of useful educational programmes published as narratives. In addition, only peer-reviewed literature was searched, and grey literature was excluded, which may also have led to the exclusion of relevant studies [75].

The included studies in this review were mainly from high-income countries, and the majority were from the US. Differences may exist in the various healthcare systems, cultural values and understanding of the concept patient- and family-centred care across cultures. Patient- and family-centred care including shared decision-making is a Western concept originated in the US [2].The study from China included in this scoping review described cultural barriers that hinder family-centred care, such as challenges with shared-decision making and family structure [62].

Finally, another limitation is that scoping reviews are highly broad and therefore can lack focus. The scoping review format does not require an appraisal of the studies included, making it difficult to compare the included studies [15, 16]. However the advantage of the scoping review is to identify knowledge gaps and set research agendas [16].

## Conclusion

This scoping review showed that there is a large variety of educational programmes in paediatric patient- and family-centred care. The educational content mainly focused on communication and relational competencies, and experiential learning was the most used educational strategy. In future research there is a need for more consistent measurement methods including long term evaluation, description of educational content and randomised controlled trials.

### Supplementary Information

Below is the link to the electronic supplementary material.

Supplementary file1 (PDF 458 KB)

## Data Availability

For charting see Online Resource 4.

## References

[1] Care CO, Patient IF, Care FC (2012). Patient-and family-centered care and the pediatrician’s role. Pediatrics.

[2] Jolley J, Shields L (2009). The evolution of family-centered care. J Pediatr Nurs.

[3] Priddis L, Shields L (2011). Interactions between parents and staff of hospitalised children. Paediatr Nurs.

[4] Bray L et al (2023) Developing rights-based standards for children having tests, treatments, examinations and interventions: using a collaborative, multi-phased, multi-method and multi-stakeholder approach to build consensus. Eur J Pedia 1–15 10.1007/s00431-023-05131-9 PMC10587267 37566281

[5] Shields L, Zhou H, Pratt J, Taylor M, Hunter J, Pascoe E (2012) Family-centred care for hospitalised children aged 0–12 years. Cochrane Database Syst Rev 10 10.1002/14651858.CD004811.pub3 PMC11531909 23076908

[6] Shields L, Zhou H, Taylor M, Hunter J, Munns A, Watts R (2012). Family-centred care for hospitalised children aged 0–12 Years: A systematic review of quasi-experimental studies. JBI Evidence Synthesis.

[7] Shields L, Pratt J, Hunter J (2006). Family centred care: a review of qualitative studies. J Clin Nurs.

[8] Segers E, Ockhuijsen H, Baarendse P, van Eerden I, van den Hoogen A (2019). The impact of family centred care interventions in a neonatal or paediatric intensive care unit on parents’ satisfaction and length of stay: a systematic review. Intensive Crit Care Nurs.

[9] Mastro KA, Flynn L, Preuster C (2014). Patient-and family-centered care. J Nurs Adm.

[10] Yu X, Zhang J (2019). Family-centred care for hospitalized preterm infants: A systematic review and meta-analysis. Int J Nurs Pract.

[11] Johnson MJ, May CR (2015). Promoting professional behaviour change in healthcare: what interventions work, and why? A theory-led overview of systematic reviews. BMJ Open.

[12] Grover S (2022). Defining and implementing patient-centered care: an umbrella review. Patient Educ Couns.

[13] Johnson A, Nguyen H, Groth M, Wang K, Ng JL (2016). Time to change: A review of organisational culture change in health care organisations. J Organ Eff.

[14] Peterson E, Morgan R, Calhoun A (2021). Improving patient-and family-centered communication in pediatrics: a review of simulation-based learning. Pediatr Ann.

[15] Peters MDJ, McInerney P, Munn Z, Tricco AC, Khalil H, Munn Z (2020). Chapter 11: Scoping Reviews (2020 version). Aromataris E.

[16] Tricco AC (2018). PRISMA extension for scoping reviews (PRISMA-ScR): checklist and explanation. Ann Intern Med.

[17] Jeppesen E, Schmidt AA, Skjoedt CK, Hybschmann J, Gjaerde LK, Hansson H, Soerensen JL. Educational programmes for paediatric healthcare professionals in patient- and family-centred care: A scoping review protocol. https://osf.io/axz7k. Accessed 24 May 2023 10.1007/s00431-024-05455-0 PMC11035470 38430279

[18] McGowan J, Sampson M, Salzwedel DM, Cogo E, Foerster V, Lefebvre C (2016). PRESS peer review of electronic search strategies: 2015 guideline statement. J Clin Epidemiol.

[19] Coyne I, Holmström I, Söderbäck M (2018). Centeredness in healthcare: a concept synthesis of family-centered care, person-centered care and child-centered care. J Pediatr Nurs.

[20] Toivonen M, Lehtonen L, Ahlqvist-Björkroth S, Axelin A (2019). Key factors supporting implementation of a training program for neonatal family- centered care - a qualitative study. BMC Health Serv Res.

[21] Toivonen M, Lehtonen L, Löyttyniemi E, Ahlqvist-Björkroth S, Axelin A (2020). Close Collaboration with Parents intervention improves family-centered care in different neonatal unit contexts: a pre-post study. Pediatr Res.

[22] Altimier L, Kenner C, Damus K (2015). The Wee Care Neuroprotective NICU Program (Wee Care): The Effect of a Comprehensive Developmental Care Training Program on Seven Neuroprotective Core Measures for Family-Centered Developmental Care of Premature Neonates. Newborn Infant Nurs Rev.

[23] Ammentorp J, Sabroe S, Kofoed PE, Mainz J (2009). Effects of a communication course for clinicians on parents' perception of care - A randomized controlled trial. Scand J Caring Sci.

[24] Ammentorp J, Kofoed OE (2010). The long-term impact of a communication course for doctors and nurses: The Parents' perspective. Commun Med.

[25] Ammentorp J, Kofoed PE, Laulund LW (2011). Impact of communication skills training on parents perceptions of care: Intervention study. J Adv Nurs.

[26] Asuncion AM, Quintos-Alagheband ML, Leavens-Maurer J, Akerman M, Janicke P, Cavanaugh S (2022). Utilization of Family as Faculty: A Patient Directed Simulation Education to Improve Patient and Family Communication during Patient-Family Centered Rounds (PFCR). Pediatr Qual Saf.

[27] Axelin A, Ahlqvist-Björkroth S, Kauppila W, Boukydis Z, Lehtonen L (2014). Nurses' perspectives on the close collaboration with parents training program in the NICU. MCN Am J Matern Child Nurs.

[28] Ayub EM, Sampayo EM, Shah MI, Doughty CB (2017). Prehospital Providers' Perceptions on Providing Patient and Family Centered Care. Prehosp Emerg Care.

[29] Blasco PA, Kohen H, Shapland C (1999). Parents-as-teachers: design and establishment of a training programme for paediatric residents. Med Educ.

[30] Bordessoule A (2022). In situ simulation training for parental presence during critical situations in PICU: an observational study. Eur J Pediatr.

[31] Borman-Shoap E (2018). Essentials of Ambulatory Care: An Interprofessional Workshop to Promote Core Skills and Values in Team-based Outpatient Care. MedEdPORTAL.

[32] Cahill H, Coffey J, Sanci L (2015). 'I wouldn't get that feedback from anywhere else': learning partnerships and the use of high school students as simulated patients to enhance medical students' communication skills. BMC Med Educ.

[33] Cardoza MP, Hood PA (2012). Comparative study of baccalaureate nursing student self-efficacy before and after simulation. Comput Inform Nurs.

[34] Cohen-Bearak A (2021). Aligning family-clinician expectations during pediatric surgical informed consent: Development and implementation of an innovative communication skills workshop. J Contin Educ Heal Prof.

[35] Czynski AJ, Souza M, Lechner BE (2021) The mother baby comfort care pathway: the development of a rooming-in-based perinatal palliative care program. Adv Neonatal Care. 10.1097/anc.0000000000000838 10.1097/ANC.0000000000000838 33783387

[36] Dean S, Long M, Ryan E, Tarnoviski K, Mondal A, Lisanti AJ (2021). Assessment of an Educational Tool for Pediatric Cardiac Nurses on Individualized Family-Centered Developmental Care. Crit Care Nurse.

[37] Fitzgerald M, Ward J (2019). Using Standardized Actors to Promote Family-centered Care. J Pediatr Nurs.

[38] Gafni-Lachter L, Ben-Sasson A (2022) Promoting family-centered care: a provider training effectiveness study. Am J Occup Ther 76(3). 10.5014/ajot.2022.044891 10.5014/ajot.2022.044891 35605168

[39] Galarza-Winton ME, Dicky T, O’Leary L, Lee SK, O’Brien K (2013) Implementing family-integrated care in the NICU: Educating nurses. Adv Neonatal Care 13(5):335–340. https://ep.fjernadgang.kb.dk/login?url=http://ovidsp.ovid.com/ovidweb.cgi?T=JS&CSC=Y&NEWS=N&PAGE=fulltext&D=emed14&AN=1372132255 10.1097/ANC.0b013e3182a14cde 24042139

[40] Gibbs D, Warren IM (2022). "Implementing infant and family-centred developmental care: Exploring the impact of an innovative educational initiative," *Acta Paediatrica*. International Journal of Paediatrics.

[41] Heginbotham L (2022). A parent-led, patient-centered medical home model instruction for interprofessional undergraduate and graduate learning opportunities. Med Educ Online.

[42] Johnson NL, Lashley J, Stonek AV, Bonjour A (2012). Children with developmental disabilities at a pediatric hospital: staff education to prevent and manage challenging behaviors. J Pediatr Nurs.

[43] Johnson AM, Yoder J, Richardson-Nassif K (2006). Using families as faculty in teaching medical students family-centered care: what are students learning?. Teach Learn Med.

[44] Kaplan BG, Holmes L, Mott M, Atallah H (2011). Design and implementation of an interdisciplinary pediatric mock code for undergraduate and graduate nursing students. Comput Inform Nurs.

[45] Katz C, Barnes M, Osta A, Walker-Descartes I (2020). The Acculturation Toolkit: An Orientation for Pediatric International Medical Graduates Transitioning to the United States Medical System. MedEdPORTAL.

[46] Keisling BL, Bishop EA, Roth JM (2017). Integrating Family as a Discipline by Providing Parent Led Curricula: Impact on LEND Trainees' Leadership Competency. Matern Child Health J.

[47] Khan A (2018). Patient safety after implementation of a coproduced family centered communication programme: multicenter before and after intervention study. BMJ.

[48] Khoo SA (2020). Improving provider-patient communication skills among doctors and nurses in the children’s emergency department. Asia Pacific Scholar.

[49] Kind T, Goldman E, Fratantoni K, Wiedermann BL, Agrawal D, Coddington D (2014). Learning to deliver care in a medical home: a qualitative analysis of residents' reflections on practice. Clin Pediatr (Phila).

[50] King G, Tam C, Fay L, Pilkington M, Servais M, Petrosian H (2011). Evaluation of an occupational therapy mentorship program: effects on therapists' skills and family-centered behavior. Phys Occup Ther Pediatr.

[51] Leaming-Van Zandt KJ, Zhu H, Banuelos RC, Lopez MA, Hsu DC (2021). Impact of a Pediatric-Focused Communication Course on Patient/Caregiver-Perceived Physician Communication Skills in a Pediatric Emergency Department. Pediatr Emerg Care.

[52] Lewis KD (2022). Patient and Family-Centered I-PASS SCORE Program: Resident and Advanced Care Provider Training Materials. MedEdPORTAL.

[53] Mandak K, Light J, McNaughton D (2020). The Effects of an Online Training on Preservice Speech-Language Pathologists' Use of Family-Centered Skills. Am J Speech Lang Pathol.

[54] McArdle GK (1994). From directive expert to non-directive partner: A study of facilitating change in the occupational self-perceptions of health visitors and school nurses. Br J Guid Couns.

[55] Meyer EC, Brodsky D, Hansen AR, Lamiani G, Sellers DE, Browning DM (2011). An interdisciplinary, family-focused approach to relational learning in neonatal intensive care. J Perinatol.

[56] Montgomery L, Benzies K, Barnard C (2016). Effects of an Educational Workshop on Pediatric Nurses' Attitudes and Beliefs About Family-Centered Bedside Rounds. J Pediatr Nurs.

[57] Mutambo C, Shumba K, Hlongwana KW (2020). Post-training and mentorship experiences of KidzAlive-trained healthcare workers at primary healthcare facilities in KwaZulu-Natal, South Africa. Afr J Prim Health Care Fam Med.

[58] Nehal US, Kanahara S, Tanabe M, Hayner G, Nelson BD (2020). Pediatric Refugee Health Care Delivery in the Community Setting: An Educational Workshop for Multidisciplinary Family-Centered Care During Resettlement. MedEdPORTAL.

[59] Newes-Adeyi G, Helitzer DL, Roter D, Caulfield LE (2004). Improving client-provider communication: evaluation of a training program for women, infants and children (WIC) professionals in New York state. Patient Educ Couns.

[60] Pawłowska M, Del Rossi L, Kientz M, McGinnis P, Padden-Denmead M (2020). Immersing students in family-centered interprofessional collaborative practice. J Interprof Care.

[61] Roter DL (2004). Use of an innovative video feedback technique to enhance communication skills training. Med Educ.

[62] Su H, Llewellyn G, Yi Y, Gao Y, Liu J (2021). The feasibility of family-centred early intervention for children with disabilities in mainland China: Practitioners' perceptions. Child.

[63] Taff H (2022). Strengthening Parent - Physician Communication: A Mixed Methods Study on Attuned Communication Training for Pediatric Residents. Teach Learn Med.

[64] Walter L, Robb M (2019). Promoting Discharge Readiness Through Staff Education: A Family-Centered Approach. J Nurses Prof Dev.

[65] Weis J, Zoffmann V, Egerod I (2014). Improved nurse-parent communication in neonatal intensive care unit: evaluation and adjustment of an implementation strategy. J Clin Nurs.

[66] Weiss S, Goldlust E, Vaucher YE (2010). Improving parent satisfaction: an intervention to increase neonatal parent-provider communication. J Perinatol.

[67] Wissow L, Gadomski A, Roter D, Larson S, Lewis B, Brown J (2011). Aspects of mental health communication skills training that predict parent and child outcomes in pediatric primary care. Patient Educ Couns.

[68] Zengin Akkus P, Ilter Bahadur E, Coskun A, Koken G, Karahan S, Ozmert EN (2020). Family-centred service: Perspectives of paediatric residents from a non-Western country. Child.

[69] Kirkpatrick D, Kirkpatrick J (2006). Evaluating training programs: The four levels.

[70] Thomas PA, Kern DE, Hughes MT, Tackett SA, Chen BY (2022). Curriculum development for medical education: a six-step approach.

[71] Abraham M, Moretz JG (2012). Implementing patient-and family-centered care: Part I-understanding the challenges. Pediatr Nurs.

[72] Clay AM, Parsh B (2016). Patient-and family-centered care: It’s not just for pediatrics anymore. AMA J Ethics.

[73] Levetown M (2008). American Academy of Pediatrics Committee on B. Communicating with children and families: from everyday interactions to skill in conveying distressing information. Pediatrics.

[74] Chesney M (2005). Comparison of child and parent satisfaction ratings of ambulatory pediatric subspecialty care. J Pediatr Health Care.

[75] Adams RJ, Smart P, Huff AS (2017). Shades of grey: guidelines for working with the grey literature in systematic reviews for management and organizational studies. Int J Manag Rev.

