# Supplementary material for: Educational programmes for paediatric healthcare professionals in patient- and family-centred care. A scoping review
Source: Eur J Pediatr. 2024 Mar 2;183(5):2015–28. doi: 10.1007/s00431-024-05455-0 (PMC11035470; doi:10.1007/s00431-024-05455-0)
Supplement: Supplementary file 1 — Supplementary file1 (PDF 458 KB) [file 431_2024_5455_MOESM1_ESM.pdf]

# Supplementary Material

## Online Resource 1

### Search strategy from PubMed

PubMED (MEDLINE): 2766 hits (March 08)

Search: ("students, health occupations"[MeSH Terms] OR "health personnel"[MeSH Terms] OR "social workers"[MeSH Terms] OR "anaesthesia assistant*"[Title/Abstract] OR "anesthesia assistant*"[Title/Abstract] OR "anaesthesiologist*"[Title/Abstract] OR "anesthesiologist*"[Title/Abstract] OR "anaesthetist*"[Title/Abstract] OR "anesthetist*"[Title/Abstract] OR "audiologist*"[Title/Abstract] OR "clinical staff"[Title/Abstract] OR "dental assistant*"[Title/Abstract] OR "dental hygienist*"[Title/Abstract] OR "dental worker*"[Title/Abstract] OR "dentist*"[Title/Abstract] OR "dietician*"[Title/Abstract] OR "dietitian*"[Title/Abstract] OR "doctor*"[Title/Abstract] OR "general practitioner*"[Title/Abstract] OR "health personnel"[Title/Abstract] OR "healthcare personnel"[Title/Abstract] OR "health professional*"[Title/Abstract] OR "healthcare professional*"[Title/Abstract] OR "healthcare provider*"[Title/Abstract] OR "healthcare worker*"[Title/Abstract] OR "hospital employee*"[Title/Abstract] OR "hospital care team*"[Title/Abstract] OR "hospital personnel"[Title/Abstract] OR "hospital staff"[Title/Abstract] OR "hospital worker*"[Title/Abstract] OR "laboratory assistant*"[Title/Abstract] OR "laboratory personnel"[Title/Abstract] OR "laboratory scientist*"[Title/Abstract] OR "laboratory technician*"[Title/Abstract] OR "medical laboratory staff"[Title/Abstract] OR "medical staff"[Title/Abstract] OR "medical student*"[Title/Abstract] OR "medical technician*"[Title/Abstract] OR "midwife"[Title/Abstract] OR "midwives"[Title/Abstract] OR "nurse*"[Title/Abstract] OR "nursing assistant*"[Title/Abstract] OR "nursing student*"[Title/Abstract] OR "nutritionist*"[Title/Abstract] OR "occupational therapist*"[Title/Abstract] OR "ophthalmic optician*"[Title/Abstract] OR "optometrist*"[Title/Abstract] OR "paramedic*"[Title/Abstract] OR "paediatric assistant*"[Title/Abstract] OR "pediatric assistant*"[Title/Abstract] OR "paediatrician*"[Title/Abstract] OR "pediatrician*"[Title/Abstract] OR "pharmacist*"[Title/Abstract] OR "pharmacy technician*"[Title/Abstract] OR "phlebotomist*"[Title/Abstract] OR "physician*"[Title/Abstract] OR "physiotherapist*"[Title/Abstract] OR "physical therapist*"[Title/Abstract] OR "porter*"[Title/Abstract] OR "psychiatrist*"[Title/Abstract] OR "psychologist*"[Title/Abstract] OR "psychotherapist*"[Title/Abstract] OR "radiographer*"[Title/Abstract] OR "radiologic technologist*"[Title/Abstract] OR "radiologist*"[Title/Abstract] OR "radiology assistant*"[Title/Abstract] OR "radiology personnel"[Title/Abstract] OR "social worker*"[Title/Abstract] OR "speech therapist*"[Title/Abstract] OR "specialist*"[Title/Abstract] OR "surgeon*"[Title/Abstract] OR "undergraduate*"[Title/Abstract]) AND ("educational status"[MeSH Terms] OR "education"[MeSH Terms] OR "professional competence"[MeSH Terms] OR "case-based learning"[Title/Abstract] OR "competence*"[Title/Abstract] OR "course*"[Title/Abstract] OR

"curricula*"[Title/Abstract] OR "curriculums*"[Title/Abstract] OR "didactic*"[Title/Abstract] OR "education*"[Title/Abstract] OR "formal learning"[Title/Abstract] OR "learning session*"[Title/Abstract] OR "paedagogic*"[Title/Abstract] OR "pedagogic*"[Title/Abstract] OR "seminar*"[Title/Abstract] OR "simulation*"[Title/Abstract] OR "skill*"[Title/Abstract] OR "teaching method*"[Title/Abstract] OR "teaching model*"[Title/Abstract] OR "teaching program*"[Title/Abstract] OR "teaching session*"[Title/Abstract] OR "training method*"[Title/Abstract] OR "training program*"[Title/Abstract] OR "training session*"[Title/Abstract] OR "training simulation*"[Title/Abstract] OR "tutorial*"[Title/Abstract] OR "workshop*"[Title/Abstract]) AND ("family nursing"[MeSH Terms] OR "patient centered care"[MeSH Terms] OR "child-centred"[Title/Abstract] OR "child-centered"[Title/Abstract] OR "client-centred"[Title/Abstract] OR "client-centered"[Title/Abstract] OR "family-centred"[Title/Abstract] OR "family-centered"[Title/Abstract] OR "family focused care"[Title/Abstract] OR "family integrated care"[Title/Abstract] OR "family nursing"[Title/Abstract] OR "parent-partnered"[Title/Abstract] OR "partnership*"[Title/Abstract] OR "patient and family centred care"[Title/Abstract] OR "patient and family centered care"[Title/Abstract] OR "patient and family engaged care"[Title/Abstract] OR "patient-centred"[Title/Abstract] OR "patient-centered"[Title/Abstract] OR "patient-directed care"[All Fields] OR "personalized care"[All Fields] OR "personalised care"[All Fields] OR "person-centred"[All Fields] OR "person-centered"[All Fields] OR "person-focused"[All Fields] OR "relationship-based"[All Fields] OR "relationship-centred"[All Fields] OR "relationship-centered"[All Fields] OR "user-centred"[All Fields] OR "user-centered"[All Fields]) AND ("child"[MeSH Terms] OR "pediatrics"[MeSH Terms] OR "adolescen*"[Title/Abstract] OR "child*"[Title/Abstract] OR "pediatric*"[Title/Abstract] OR "paediatric*"[Title/Abstract] OR "neonat*"[Title/Abstract])

**Online Resource 2**

**Keywords**

| | ***Population*** | ***Concept*** | ***Context*** | |
|---|---|---|---|---|
| MeSH | Health occupations students | Education | Family nursing | Child |
| | Health Personnel | Professional competence | Patient-centered care | Pediatrics |
| | Social workers | | | |
| | | | | |
| Title/Abstract | An?esthesia assistant* | Case-based learning | Child-cent?red | Adolescen* |

| | | | | |
|---|---|---|---|---|
| | An?esthesiologist* | Competence* | Client-cent?red | Child* |
| | An?esthetist* | Course* | Family-cent?red | Neonat* |
| | Audiologist* | Curricula* | Family focused Care | P?ediatric* |
| | Clinical staff | Curriculum* | Family integrated Care | |
| | Dental assistant* | Didactic* | Family nursing | |
| | Dental hygienist* | Education* | Parent-partnered | |
| | Dental worker* | Formal learning | Partnership* | |
| | Dentist* | Learning session* | Patient- and family-cent?red care | |
| | Dietician* | P?edagogic* | Patient- and Family-engaged care | |
| | Dietitian* | Seminar* | Patient-cent?red | |
| | Doctor* | Simulation* | Patient-directed care | |
| | General practitioner* | Skill* | Personali?ed care | |
| | Health personnel | Teaching method* | Person-cent?red | |
| | Healthcare personnel | Teaching model* | Person-focused | |
| | Health professional* | Teaching program* | Relationship-based | |
| | Healthcare professional* | Teaching session* | Relationship-cent?red | |
| | Healthcare provider* | Training method* | User-cent?red | |
| | Healthcare worker* | Training program* | | |
| | Hospital employee* | Training session* | | |
| | Hospital care team* | Training simulation* | | |
| | Hospital personnel | Tutorial* | | |

| | | | | |
|---|---|---|---|---|
| | Hospital staff | Workshop* | | |
| | Hospital worker* | | | |
| | Laboratory assistant* | | | |
| | Laboratory personnel* | | | |
| | Laboratory scientist* | | | |
| | Laboratory technician* | | | |
| | Medical laboratory staff | | | |
| | Medical staff | | | |
| | Medical student* | | | |
| | Medical technician* | | | |
| | Midwife | | | |
| | Midwives | | | |
| | Nurse* | | | |
| | Nursing assistant* | | | |
| | Nursing student* | | | |
| | Nutritionist* | | | |
| | Occupational therapist* | | | |
| | Ophthalmic optician* | | | |
| | Optometrist* | | | |
| | Paramedic* | | | |
| | P?ediatric assistant* | | | |
| | P?ediatrician* | | | |
| | Pharmacist* | | | |
| | Pharmacy technician* | | | |
| | Phlebotomist* | | | |
| | Physician* | | | |
| | Physiotherapist* | | | |
| | Physical therapist* | | | |
| | Porter* | | | |
| | Psychiatrist* | | | |
| | Psychologist* | | | |
| | Psychotherapist* | | | |

| | | | | |
|---|---|---|---|---|
| | Radiographer* | | | |
| | Radiologic technologist* | | | |
| | Radiologist* | | | |
| | Radiology assistant* | | | |
| | Radiology personnel | | | |
| | Social worker* | | | |
| | Speech therapist* | | | |
| | Specialist* | | | |
| | Surgeon* | | | |
| | Undergraduate* | | | |

**Online Resource 3 Data coding sheet**

| Citation | Year | Country | Type of healthcare professionals/ n | Paediatric population | Duration | Educational strategy | Educational content | Educational objectives | Needs assessed locally | Study design | Assessment method | Findings/main outcomes | Theoretical framework | Accreditation of programmes | Kirkpatrick level |
|---|---|---|---|---|---|---|---|---|---|---|---|---|---|---|---|
| | | | | | | | | | | | | | | | |
| | | | | | | | | | | | | | | | |
| | | | | | | | | | | | | | | | |
| | | | | | | | | | | | | | | | |
| | | | | | | | | | | | | | | | |

## Online Resource 4

| Citation | Year | Country | Type of healthcare professionals/ n | Paediatric population | Duration | Educational strategy | Educational content | Educational objectives | Needs assessed locally | Study design | Assessment method | Findings/ main outcomes | Theoretical framework | Accreditation of programmes | Kirkpatricks level |
|---|---|---|---|---|---|---|---|---|---|---|---|---|---|---|---|
| Altimier L, Kenner C, Damus K. The Wee Care Neuroprotective NICU Program (Wee Care): The Effect of a Comprehensive Developmental Care Training Program on Seven Neuroprotective Core Measures for Family-Centered Developmental Care of Premature Neonates Newborn and Infant Nursing Reviews 2015;15(1):6-16 | 2015 | USA/Belgium/the Netherlands | All NICU staff (Nurses, neonatal nurse practitioners, physicians, occupational, physical, respiratory & speech therapists, nursing assistants/technicians, lactation consultants, pharmacists, social workers, case managers/discharge planners, unit secretaries, housekeepers, x-ray technicians, and unit assistants) (N=81 NICU sites, it is not stated how many staff members were educated) | NICU (neonatal intensive care unit) | 18 months | The Wee Care Neuroprotective NICU program (Philips.Healthcare). On-line e-learning, didactic education and hands-on interactive workshop for staff. Leadership workshop with action plans and goals. Goal attainment monitored monthly, through team meetings (phone or on-site) . Educators worked with staff minimum 1 year to ensure goal attainment. On-site clinical and leadership follow-up visits, at various intervals throughout the implementation year. | Healing Environment, Partnering with Families, Positioning and Handling, Safeguarding Sleep, Minimizing Stress and Pain, Protecting Skin, and Optimizing Nutrition | Optimise the NICU environment and caregiving practices in order to facilitate the best outcomes for premature infants and their families | Yes | Pretest-Posttest Design | Wee Care survey tool, 4-8 weeks prior program and 12-14 months after the training intervention were implemented. Assessed by educator/consultant | Significantly improving seven neuroprotective core measures for family-centred developmental care, 28% increase for Partnering with Families | Neuroprotective family-centred developmental care, transformational change methodology | n/a (standardised program developed in 1997, owned by Philips Healthcare) | 3 |

| | | | | | | | | | | | | | | |
|---|---|---|---|---|---|---|---|---|---|---|---|---|---|---|
| Ammentorp J, Sabroe S, Kofoed PE, Mainz J. Effects of a communication course for clinicians on parents' perception of care - A randomized controlled trial Scandinavian Journal of Caring Sciences 2009;23(3):506-517 | 2009 | Denmark | Doctors (N=10), nurses (N=5) | Outpatients | 5 days (3+2 days separated by 4 weeks) | Education in patient-centred communication by a paediatrician 3 days, then 4 weeks rehearsal by participants and video recordings of consultations, then 2 days feedback on videos | Communication and patient-centred perspective: Eliciting and understanding patient concerns and needs, reaching a shared understanding of the problem and its treatment were the overall issues. Content focused on the structure of the consultation (how to secure that every relevant aspect was considered and that overview and transparency were ensured?). Communication techniques on how to listen, how to help the patient to formulate the problems, how to ask the right questions. | Increase patient-centred communication | No | Randomised controlled trial | Questionnaire developed to the study based on a validated questionnaire answered by parents pre course 271 and post 764 | No significant difference in parents satisfaction and perception of communication . | Theory of social learning, patient-centred communication, patient-centred care, | n/a (offered by the Danish Medical Association) | 4 |
| Ammentorp, J.; Kofoed, O. E. The long-term impact of a communication course for doctors and nurses: The Parents' perspective. Communication and Medicine 2010;7(1):3-10 | 2010 | Denmark | Doctors (N=21), nurses (N=13) | Outpatients | 5 days (3+2 days separated by 4 weeks) or 3 days (2+1 day separated by 4 weeks) | Lectures first course days. Then 4 weeks practising and video recording of own consultation. Last course day(s) feedback and discussion | Communication: Tight structure of the consultation, communication techniques on how to listen, how to help a patient to formulate their problems and how to ask the right questions etc; A patient-centred approach that facilitates a broader understanding of the patients´concerns and needs and a shared understanding of the problem and its solution. | Improve communication skills | Yes | Pretest-Posttest Design | Questionnaire developed to the study based on a validated questionnaire answered by parents, administered 1-1157 days after the course | The proportion of satisfied parents increased significantly, evenly distributed over time from course, the greatest improvements occurred in relation to the statement: "the clinician tried to understand how I experienced the problem" | Patient-centred communication, social learning, patient-centred approach, | n/a (certified by the Danish Medical Association) | 4 |

| | | | | | | | | | | | | | | | |
|---|---|---|---|---|---|---|---|---|---|---|---|---|---|---|---|
| Ammentorp J, Kofoed PE, Laulund LW. Impact of communication skills training on parents perceptions of care: Intervention study Journal of Advanced Nursing 2011;67(2):394-400 | 2011 | Denmark | Nurses (N=26), physicians (N=4), psychologist (N=1), hospital teacher (N=1) | Paediatric inpatients | 3 days (2+1 day separated by 4 weeks) | 2 days with lectures, 4 weeks practising and video recording of own consultation, 1 day feedback and discussion | Communication skills & patient-centred approach: Tight structure of the consultation with reference to The Calgary-Cambridge Guide, communication techniques: How to listen, how to help a patient to formulate their problems, how to ask the right questions, how to elicit and respond to patient concerns and needs, and how to reach a mutual understanding of the problem and its treatment. | Improve communication skills | No | Pretest-Posttest Design | Electronic questionnaire developed to the study, 22 questions, answered by parents, pre course 895 and post 1937 | The proportion of satisfied parents icreased significantly on four questions, this was about continuity, consistent information,suf ficient time and staff understanding their situation. For the information questions, no significant differences were found. | Patient-centred care, patient-centred communication, Theory of social learning | n/a (offered by the Danish Medical Association) | 4 |
| Asuncion AM, Quintos-Alagheband ML, Leavens-Maurer J, Akerman M; Janicke P, Cavanaugh S. Utilization of Family as Faculty: A Patient Directed Simulation Education to Improve Patient and Family Communication during Patient-Family Centered Rounds (PFCR). Pediatr Qual Saf May-Jun 2022;7(3):e551 | 2022 | USA | Paediatric housestaff (N=36), attending hospitalists (N=6), nurses (N=?) | Inpatients (general) | n/a | In-situ simulation: Child mannequin and faculty member as parent. 2 cases: a 4-week-old infant with fever and a 7-year-old child with asthma. Simulated patient- and family-centred rounds, debiefing afterwards | 2 cases (4-week-old infant with fever and a 7-year-old child with asthma). Greet and introduce the primary care team, establish partnership with the family, explain the medical condition in plain language, family opportunity to ask questions, communication model of partnership, included and engaged nursing, | Improve medical providers' communication | Yes | Pretest-Posttest Design | Observation of bedside rounds before and after simulation using checklist (3 observers) and national standarised, public reported survey: The Hospital Consumer Assessment of Healthcare Providers and Systems (HCAHPS). | Patient Experience of Care in Physician communication with family measured by HCAPHS improved from baseline 72% to 82%.Observers checklist: Significant improvement in 4 of 6 competencies (1. greet and introduce the primary team 2. establish partnership 3. explain medical condition 4. include and engage nursing). | Patient-family-centred care | n/a | 3+4 |

| | | | | | | | | | | | | | | |
|---|---|---|---|---|---|---|---|---|---|---|---|---|---|---|
| Axelin A, Ahlqvist-Björkroth S, Kauppila W, et. al.<br>Nurses' perspectives on the close collaboration with parents training program in the NICU<br>MCN Am J Matern Child Nurs Jul-Aug 2014;39(4):260-8 | 2014 | Finland | The entire NICU staff: Nurses (N=45), physicians (N=4), physiotherapist (N=1) | NICU (neonatal intensive care unit) | 3,5 years (2 years for mentors and 18 months for tutored group) | Close Collaboration with Parents Training Program: Mentors group (half of the staff): 1 day of lectures + a week of small group demonstrations for each theme(4 themes), six individual mentoring sessions, and regularly supervision groups. The tutored group: same amount hands-on teaching, and reflective supervision, but reading written material instead of lectures. | Observing preterm infant behavior, watching babies with parents, understanding individual features of families, and family-centered discharge planning | Educate caregivers regarding a change to family-centred care/ to teach nurses and physicians to collaborate closely with parents in infant care. | Yes | Qualitative | Focus groups with semi-structured interviews with 22 nurses | Changed staff attitudes and care practices | Family-centred care, multimethod learning philosophy, neurobehavioral theory, attachment theory | n/a | 1+2+3 |

| | | | | | | | | | | | | | | | |
|---|---|---|---|---|---|---|---|---|---|---|---|---|---|---|---|
| Ayub EM, Sampayo EM, Shah MI, Doughty CB. Prehospital Providers' Perceptions on Providing Patient and Family Centered Care Prehosp Emerg Care Mar-Apr 2017;21(2):233-241 | 2017 | USA | Emergency Medical Technicians and paramedics (N=122) | Simulated paediatric emergencies in the prehospital setting | 1 day (not stated clearly in the article) | Paediatric Simulation Training for Emergency Prehospital Providers (PediSTEPPS): didactic lecture, paediatric skills stations, and four high fidelity simulation scenarios with actors as parents, followed by a scripted debriefing session | Content of didactic lecture not described. Simulation scenarios: Case of respiratory distress/failure or non-accidental trauma (the two last scenarios not described), the actors were trained to respond naturally to how the personnel interacted with them | To provide simulation-based education for prehospital providers caring for critically ill children in the prehospital environment | No | Qualitative | Audio recordings from debriefing sessions, member checking by two focus group interviews | Four main themes: (1) Perceived barriers to providing patient- and family centred care (2) Providing emotional support to caregivers (3) Strategies for effective communication (4) Tactics to overcome perceived caregiver barriers. | Patient- and family centred care, grounded theory, | n/a , though a standardised program (PediSTEPPS) | 2 |
| Blasco PA, Kohen H, Shapland C. Parents-as-teachers: design and establishment of a training programme for paediatric residents. Med Educ. 1999 Sep;33(9):695-701. | 1999 | USA | Paediatric residents (N=18) | Children with chronic disabling conditions | 25 minutes video + discussion, 2x2-4 hours visit with a family, 90 minutes debriefing | "Parents-as-teachers"( PAT): a video with parent experiences and discussion, 2 visits with a family with a disabled child, a debriefing session, letter to the parents | Video: parent experiences with the health care system in relation to their child with a developmental disability. Visits: Learn through home and community site visits about the importance of developing partnerships with parents and service providers in order to better care for children with chronic conditions | Improve the training of paediatric residents in developmental disabilities in general and to provide the residents more meaningful insights into family functioning | Yes | Cross-sectional | Written and oral feedback on likert scale 1-5 about quality of teaching, quality of experience and processing discussion and in addition spontaneous comments. Families rated their experiences on likert scale 0-10 and spontaneous comments. | Residents: Qualtiy of experience 4.6; quality of teaching 4.4; processing discussion 4.0, (5=excellent). Parents ratings 7.4 of 10 (10=wonderful) . Comments mostly about logistics otherwise mainly positive (both parents and residents). | Family-centred care | n/a | 1 |

| | | | | | | | | | | | | | | | |
|---|---|---|---|---|---|---|---|---|---|---|---|---|---|---|---|
| Bordessoule A, Felice-Civitillo C, Grazioli S; Barcos F, Haddad K, Rimensberger PC, Polito A. In situ simulation training for parental presence during critical situations in PICU: an observational study. Eur J Pediatr Jun 2022;181(6):2409-2414 | 2022 | Switzerland | Physicians (N=44), nurses (N=127), nursing assistants (N=28), medical students (N=2) | PICU (Paediatric Intensive Care Unit) | A half day | Simulation: Human patient simulator (Simkids, Laerdal Medical), 2 actors playng the parents, 4 different scenarios: hemorrhagic shock; sei zures; accidental extubation; tricyclic acid intoxication.Groups: 2 physicians, 3 nurses, 1 nursing assistant. (One scenario for each group). Post-event debriefing. | Family presence during a critical situation, focus on communication skills, two actors playing the parental role, to simulate family presence during CPR or other critical situation, pre-planned parental behavior (culpability, anger, despair, aggressiveness, physical and emotional breakdown) was performed by the actors, deliberately disrupted patient care | Teaching the communication skills necessary to manage family presence during critical situations or resuscitation | No | Cross-sectional (pre results collected in post educational survey) | Questionnaire created by the simulation team, rating scale 0-10, administered immediately after debriefing session. | Perceived stress associated with parental presence decreased from pre 6 (IQR, 4–7) to 4 (IQR, 2–5) ($p < 0.0001$). 25.7% perceived post-simulation stress level was higher than pre. Satisfaction of the participants was high with a median of 10 (IQR, 9–10) | Family-centred approach, patient- and family-centred care, family-centred care, | n/a | 1+2 |

| | | | | | | | | | | | | | | | |
|---|---|---|---|---|---|---|---|---|---|---|---|---|---|---|---|
| Borman-Shoap E, King, E.; Hager K, Adam P, et. al. Essentials of Ambulatory Care: An Interprofessional Workshop to Promote Core Skills and Values in Team-based Outpatient Care MedEdPORTAL May 4 2018;14():10714 | 2018 | USA | Nurse practitioner graduate students (N=99), internal medicine residents (N=67), family medicine residents (N=24), medicine-paediatric residents (N=24), paediatric residents (N=46), pharmacy residents (N=69), (24-51 learners in each workshop, interdisciplinary) | Outpatients | 1 day | Workshop: PowerPoint, handouts, discussion, brainstorming sessions, small group activities, role play, quiz, | Teamwork, conflict management, systems thinking, Swiss cheese model, the patient-centered health care home, and patient-centered communication (observing, and practicing patient-centered communication skills, including the skill of agenda setting at the start, the art of polite interruption in the middle, and the technique of cocreating a plan to close the visit), | 1. Define the characteristics of effective teams.<br>2. Compare and contrast the roles of various team members in the outpatient clinic setting.<br>3. List the elements of the patient-centred health care home.<br>4. Apply systems theory to the design of an outpatient clinic space to meet the goals of the Triple Aim.<br>5. Deploy the Patient-Centred Observation Form to enhance patient-centred communication skills. | Yes | Cross-sectional (pre results collected in post educational survey) | Self-designed survey with 5-point Likert scale | Higher self-perceived level of knowledge in patient-centred interviewing, patient-centred healthcare home, communication techniques, team-based care and systems thinking in patient-centred care delivery, a majority would use the concepts in their practice, | Patient-centred care, patient-centred communication, patient-centred healthcare home model, systems theory | n/a | 2 |
| Cahill H, Coffey J, Sanci L. 'I wouldn't get that feedback from anywhere else': learning partnerships and the use of high school students as simulated patients to enhance medical students' communication skills. BMC Med Educ. 2015 Mar 7;15:35. | 2015 | Australia | Medical students in the fifth year (N=170) | High school students (15-16 years) | 2 hours | Workshop ("Learning Partnerships"): The medical student play the family physician and use HEADSS psychosocial screening tool, the student play a fictional character, the adolescents participate both as actors and coaches (providing feedback after each phase of the screening). The proces is co-facilitated by a classroom teacher and a medical educator | Learning Partnerships, communication-training, HEADSS-model framework for questions, key skills such as making the confidentiality statement, arranging for time alone with the adolescent patient, use of suitably framed normalising or non-judgemental smorgasbord questions, and explaining the purpose of the screening | Enhance medical students' communication skills | Yes | Mixed-methods pre-post study | Pre-post focus group, semi-structured interview, post workshop survey | Improved skills and confidence in communicating with adolescents about sensitive health issues. Thought it was valuable for improving skills | Experiential learning, "learning partnerships", HEADSS model | n/a | 1+2 |

| | | | | | | | | | | | | | | | |
|---|---|---|---|---|---|---|---|---|---|---|---|---|---|---|---|
| Cardoza, M. P.; Hood, P. A. Comparative study of baccalaureate nursing student self-efficacy before and after simulation. Comput Inform Nurs Mar 2012;30(3):142-7 | 2012 | USA | Senior baccalaureate nursing students (N=52) | Paediatric manikin | 7 weeks | The first and last day: Simulation x2 with paediatric manikin, parent actor, student performances videotaped, debriefing & learning modules. In the weeks between: Paediatric lectures and 5 hospital based clinical paediatric shifts | Simulation (acute appendix, sepsis, asthma, multiple trauma/end of life), parent actors, learning modules that included cardiopulmonary resuscitation, therapeutic communication, pediatric growth, development reviews | Build self-efficacy and confidence in providing family-centred care | No | 2x Pretest-Posttest Design | Participan self-efficacy measured with GSE scale (General Self-Efficacy scale) | Contradictory results | Social cognitive theory, family-centred care, experiential learning | n/a | 2 |
| Cohen-Bearak A, Meyer EC, Mednick L, Varrin P, Burgess L, Kuhlmann PH, Bell S, Lillehei C. Aligning Family-Clinician Expectations During Pediatric Surgical Informed Consent: Development and Implementation of an Innovative Communication Skills Workshop. J Contin Educ Health Prof. 2021 Oct 1;41(4):279-285. | 2021 | USA | Surgeons (N=30) and nurse practitioners (N=5) | Urology and general surgery | 2 hours + 20 minutes 2 months later | Workshop: Introduction, film clip, discussion, presentation of literature review and qualitative results, enactment with actors and clinicians and debriefing, discussion of strategies, education on how to discuss surgery with children, final reflections, take-homes and evaluation. Booster session with highlights. | Communication, parent as partners, relational skills: Five critical ways to enhance communication during surgical consent discussions: Customize communication, align expectations, share clinical uncertainty, recognize and attend to emotions, identify team members. | To improve the communication between surgical staff and paediatric patients/families during the surgical informed consent process | Yes | Mixed methods | Postworkshop questionnaires and booster session feedback | 3/4 reported the learning to be valuable, and 64% were likely to change practice. 87% percent would recommend the workshop to other colleagues, and 58 to 74% felt more prepared to achieve each of eight specific skills. After booster session several participants reported changed practice | Relational learning, PERCS (Program to Enhance Relational Comunication Skills), family-centred | n/a | 1+2+3 |

| | | | | | | | | | | | | | | |
|---|---|---|---|---|---|---|---|---|---|---|---|---|---|---|
| Czynski AJ, Souza M, Lechner BE. The Mother Baby Comfort Care Pathway: The Development of a Rooming-In-Based Perinatal Palliative Care Program. Adv Neonatal Care. 2021 Mar 25. | 2021 | USA | Nurses (N=30) | Infants with a lethal diagnosis | 2 hours | Workshop with lecture. (The education was part of a program to facilitate change: The Mother Baby Comfort Care Pathway) | Background of perinatal palliative care, practical provision of neonatal palliative care, communication skills, provision of end-of-life care, provision of bereavement care, memory making | Facilitating a family-centered postpartum experience to those newborns and their families that are facing life-limiting illnesses. | Yes | Cross-sectional | Post educational survey: regarding experience of workshop | The nurses appreciated the workshop (N=7) and it facilitated implementation (N=3) | Family-centred care | n/a | 1+2 |
| Dean S, Long M, Ryan E, Tarnoviski K, Mondal A, Lisanti AJ. Assessment of an Educational Tool for Pediatric Cardiac Nurses on Individualized Family-Centered Developmental Care. Crit Care Nurse. 2021 Apr 1;41(2):e17-e27. . | 2021 | USA | Paediatric cardiac inpatient nurses (N=290) and procedural nurses (N=40) | Infants born with congenital heart disease | n/a | Visual educational tool: Developmental care flowers. One-on-one education or staff huddles | Content displayed on the inpatient and procedural Development Care Flowers. Cue-based care, environment, patient positioning, family-centred care, induvidualised care, support inpatient developmental care | (1) increase nurses' self reported knowledge of IFDC (Individualized Family-Centred Developmental Care) and (2) decrease nurses' perception of barriers to IFDC. | Yes | Pretest-Posttest Design | Pre-post surveys (15-40% response rate) | Many practices, beliefs and barriers didn't change significantly, though i.e. allowing parents to be present for procedures did. It improved understanding (41% inpatient, 81% procedural), helped to integrate developmental care interventions (39% inpatient, 87% of procedural). Qualitative | Individualized family-centred developmental care | n/a | 1+2+3 |

| | | | | | | | | | | | | | | | |
|---|---|---|---|---|---|---|---|---|---|---|---|---|---|---|---|
| | | | | | | | | | | | | feedback was positive | | | |
| Fitzgerald M, Ward J.Using Standardized Actors to Promote Family-centered CareJ Pediatr Nurs Mar-Apr 2019;45():20-25 | 2019 | USA | Nursing students (N=146) | Paediatric mannequin (child with asthma, infant with fever) | 15 minutes acting, duration of introduction & debriefing not stated | Students paired, one interacted with actor + paediatric mannequin, and the other observed. Written report about the patient before entering. Background information + a few questions to think about on the door. Both students attended debriefing afterwards | Simulation (scenarios: asthma and fever). Actress asked challenging questions to train how to respond to the caregiver, verbal and nonverbal therapeutic communication techniques, responding in an empathic manner. Debriefing: What do you think this exercise was looking for? For what reason is this important? How would you use this information? Take away message: Treat every patient and familymember as you would want to be treated, as if you can understand how they are feeling. | Simulation: To enhance empathic communication and nursing skills with children and their families. Debriefing: Evaluate to facilitate nursing care of children and families | No | Mixed method design | Jefferson Scale of Patient Perception of the Health Professional's Empathy (JSPPHPE), Qualitative data analysis from debriefing sessions | Peers wieved students significant more empathetic than actors. Thematic analysis indicated that empathy needs to extend beyond the patient to the family. | Family-centred care, experiential learning theory | n/a | 2 |

| | | | | | | | | | | | | | | |
|---|---|---|---|---|---|---|---|---|---|---|---|---|---|---|
| Gafni-Lachter L, Ben-Sasson A. Promoting Family-Centered Care: A Provider Training Effectiveness Study. Am J Occup Ther May 2022;76(3) | 2022 | Israel | Occupational therapists (N=68), speech-language pathologists (N=9), physical therapists (N=2), art therapists (N=3) | Outpatients, clinic setting and school based practice | 5 hours x 6 over 10 weeks | "Better Together (BT) training program": 6 content modules based on core knowledge and skills in family-centred care. Interactive lectures with group discussion, experiential (workplace) learning tasks, reflective assignments with instructor feedback, peer mentoring tasks and individualised action plans | Principles, evidence, learning from families, development of cultural competence, strategies for effective communication with families: listening and sharing information, collaborative goal setting, family-centred care processes and measures, and promotion of it in the workplace | To promote providers' skills and self-efficacy in delivering family-centred care | No | Pretest-Posttest Design | 2 validated questionnaires by participants pre and post program: Measure of Processes of Care-Service Provider (MPOC), Measure of Processes of Care-Confidence (MPOC-Con). Satisfaction report sent electronically after end of program. | Significant changes in 3 of 4 domains of MPOC–SP and in all domains of MPOC-Con. Participants reported high satisfaction overall, but only 51.2% responded to the satisfacion report. | Family-centred care, adult learning theory, self-efficacy, | n/a | 1+2+3 |
| Galarza-Winton ME, Dicky T, O'Leary L, et al. Implementing family-integrated care in the NICU: Educating nurses Advances in Neonatal Care October 2013;13(5)():335-340 | 2013 | Canada | Nurses (N=35) | NICU (neonatal intensive care unit) | 4 hours | Workshop: Simon and Chabris' "invisible gorilla" video, research and theory, case studies and role playing, parent advocates led discussions reflecting their own NICU experiences. (Part of program (FICare) which included parent education and peer-to-peer support for parents). | Enhance communication and coaching skills: Understanding the parent experience, recognizing parents' psychological challenges, addressing the challenges of developing a therapeutic relationship with parents, providing developmentally appropriate care for newborns, understanding the therapeutic impact of the parent-infant relationship on infant outcomes, supporting parental competency, overall family functioning | (1) understanding the parent experience in the NICU (2) recognizing parents' psychological challenges (3) addressing the challenges of developing a therapeutic relationship with parents (4) providing developmentally appropriate care for newborns and understanding the therapeutic impact of the parent-infant relationship on infant outcomes; (5) supporting parental competency and overall family functioning. | Yes | Qualitative | Structured interview one-on-one, 6 months after workshop | Workshop prepared them to provide family-integrated care,most useful topics: 1) information on nursing versus parental responsibilities 2) the parent experience in the NICU 3) developmental care strategies. Identified need for ongoing staff mentoring. | Family-centred care, family-integrated care, care-by-parent model | n/a | 1 |

| | | | | | | | | | | | | | | |
|---|---|---|---|---|---|---|---|---|---|---|---|---|---|---|
| Gibbs D, Warren I. Implementing infant and family-centred developmental care: Exploring the impact of an innovative educational initiative. Acta Paediatrica, International Journal of Paediatrics 2022;(): | 2022 | UK | Nurses (N=2), allied health professionals (occupational therapist, physiotherapist) (N=5) | NICU (neonatal intensive care unit) | 6 months | "The Family and Infant Neurodevelopmental Education (FINE) programme": Orientation meeting, self-directed study (5 hours) that includes reading material, observation assignment and reflective writing, sent to a mentor for review (x10), evaluation meeting | Infant communication, increase sensitivity to infant, advocates for infants and families, understanding of developmental needs of preterm and other new-born infants, confidence in sharing knowledge, "read the baby", aware of own strengths and challenges, evaluation of current practice and identify possibilities of change | To build skills and develop role models for good practice with an individualised approach to infant- and family-centred developmental care. | No | Qualitative | Individual semistructured interviews at three timepoints (at the beginning, at completion and 6 months following programme). | Positive outcome on knowledge and skills, their performance of their clinical role and the influence on their relationships with other neonatal staff. | Infant and family-centred developmental care | n/a | 1+2 |
| Heginbotham, L.; Baugh, G. et .al A parent-led, patient-centered medical home model instruction for interprofessional undergraduate and graduate learning opportunities Med Educ Online Dec 2022;27(1):2012105 | 2022 | USA | Undergraduate (senior) Nursing Community Health, third-year Pharmacy, third year undergraduate medicine, and medical residents in Paediatrics (N=65) | Children with special healthcare needs | 3.5 hours (including 1 hour family mentor training) | Reading materials prior + in-person workshop (video, discussion, breakout sessions with parent mentors, final group reflection) | Reading material: 'Patient- and Family-Centered Care and the Pediatrician's Role', A reference to People First Language, Questions for the team-based activity. Workshop: Video 3 families of children with special health-care needs daily life experiences (experiences with their children's health condition, day-to-day schedules, experiences with health-care providers, final points each family thought was important for providers to know when establishing health-care | Increase knowledge and perceived skills within the context of children with special health-care needs and their care | No | Pretest-Posttest Design | Baseline and post-workshop survey with Likert Scale 1-5, up to one week before and one week after workshop | Learner knowledge, awareness of professional role, and perception of importance of medical specialist and nurses significantly increased. Average ratings of elements of program between 3.93 to 4.56. | Patient-centred medical home (PCMH) model, patient-centred care, family-centred care | n/a | 1+2 |

| | | | | | | | | | | | | | | | |
|---|---|---|---|---|---|---|---|---|---|---|---|---|---|---|---|
| | | | | | | | regimens), the importance of the patient-centred medical home (PCMH), the components of PCMH, questions for the family mentors, ie what is patient-centered care | | | | | | | | |
| Johnson AM, Yoder J, Richardson-Nassif K. Using families as faculty in teaching medical students family-centered care: what are students learning? Teach Learn Med Summer 2006;18(3):222-5 | 2006 | USA | Paediatric clerkship students (N=58) | Children with special healthcare needs | Duration not stated (one home visit) | A home visit in a family with a child with special healthcare needs | Home visit in which the families and children share their story of what it is like to have/be a child with special needs and introduce their family lives to the medical student | To learn family-centred care/ to support and collaborate with families who have children with special needs | No | Qualitative | Students reflection papers | 66% noted that family challenges, such as divorce or job hardships, were talked about. The 2nd and 3rd most noted reflection were the strengths within family relationships and the normalcy of the family. Physician issues most often discussed: how physicians did or did not collaborate, listen, support, or | Family-centred care | n/a | 2 |

| | | | | | | | | | | | | | | | |
|---|---|---|---|---|---|---|---|---|---|---|---|---|---|---|---|
| | | | | | | | | | | | | communicate clearly | | | |
| Johnson NL, Lashley J, Stonek AV, Bonjour A. Children with developmental disabilities at a pediatric hospital: staff education to prevent and manage challenging behaviors. J Pediatr Nurs. 2012 Dec;27(6):742-9. | 2012 | USA | Nurses, nursing assistants, student nurses, n=604 | Children with developmental disabilities | 2 hours | 1-hour online education:15-minute-long case-based lessons. 1-hour-long instructor-led class: Videos, discussions, practice of communication methods, and teaching. | 1-hour online education: An overview of developmental disabilities, preparation strategies, communication strategies, play strategies. 1 hour-long Instructor-led class: Focus on self-efficacy, family-centered care, play strategies, and communication strategies to prevent and manage challenging behaviors, 2-minute-long videos x 6 (role modeled strategies for preventing or dealing with challenging behaviors). | Prevent and manage challenging behaviours and to decrease staff fear of caring for children with developmental disabilities | Yes | Pretest-Posttest Design | Survey after on-line lesson (5-point likert scale) and pre-post in-person training (10-point likert scale) | On-line survey (n=603): knowledge and relevance rated between 4.28 to 4.48. In-person (n=42): Increased knowledge 5.5 to 8.7 and decreased fear 3.2 to 2.7 working with children with developmental disabilities | Self-efficacy theory, family-centred care, | n/a | 1+2 |

| | | | | | | | | | | | | | | | |
|---|---|---|---|---|---|---|---|---|---|---|---|---|---|---|---|
| Kaplan BG, Holmes L, Mott M, Atallah H. Design and implementation of an interdisciplinary pediatric mock code for undergraduate and graduate nursing students. Comput Inform Nurs. 2011 Sep;29(9):531-8. | 2011 | USA | Baccalaureate nursing students (N=43), emergency nurse practitioner students (N=12) | "SimBaby"( (Laerdal Medical, Wappingers Falls, NY) (a paediatric patient simulator) | 1 hour preparatory class, 20 minutes simulation, 40 minutes debriefing + time for reading in advance | One week before simulation information to read. A preparatory class before simulation. Simulation: Teams of 4-5 students, faculty member played the role of mother, infant with fever, condition worsened needing resuscitation, two emergency nurse practitioner as team leader and airway manager. Debriefing after simulation. | One week before: case information, roles, preparatory material about resuscitation, medication, communication. One hour before simulation: mock code preparatory class. Simulation: Infant with fever. A faculty member played the role of the infant's mother to include aspects of family-centred care. Debriefing among other about patient/family-centred care | Incorporate patient-/family-centred care, interprofessional teams, evidence-based practice, clinical reasoning, patient safety, and practice across the life span. | No | Pretest-Posttest Design | Pre- and post survey adressing confidence + evaluation forms post training | Increased confidence in caring for critical ill infant. Students thought the course was well organized, enjoyable, increased knowledge base and ability to function in the clinical setting and that the scenario was believable. | Patient-/family-centred care, family-centred care | n/a | 1+2 |
| Katz C, Barnes M, Osta A, Walker-Descartes I. The Acculturation Toolkit: An Orientation for Pediatric International Medical Graduates Transitioning to the United States Medical System MedEdPORTAL Jul 16 2020;16():10922 | 2020 | USA | Paediatric interns (mainly international medical graduates) (N=36) (12 in each class) | General | 1 hour x4 (1 hour x3 assesed in the study) | "The Acculturation Toolkit" consists of 4 workshops (4 presented, but just 3 evaluated in the study). Prior to each workshop reflection questions sent & participants submitted clinical scenarios. Workshops included PowerPoint, group discussions, role-play, and evaluation. | Sharing background & experiences, introduced concept patient-centered care, presented historical context for mistrust in the medical profession by certain groups, physician-patient communication (challenging patient scenarios, conflict, and de-escalation strategies), the importance of the psychosocial history, defined and discussed health | 1. Demonstrate an improved understanding of US medical culture. 2. Illustrate a shift from a doctor-centred approach to one more focused on patient-centred care. 3. Apply tools to facilitate communication in challenging patient encounters. 4. Demonstrate an increase in confidence in communicating with patients | Yes | Pretest-Posttest Design | Patient-Practitioner Orientation Scale (PPOS), pre and post workshop and questionnaire developed to the study post workshop and 1 year after. | PPOS scores revealed a significant increase in importance of patient-centred care. Satisfaction ratings for workshops was high, role play favourite activity. One year after participants assessed the workshop above moderately influential, and reported | Patient-centred care, doctor-patient communication | n/a | 1+2+3 |

| | | | | | | | | | | | | | | | |
|---|---|---|---|---|---|---|---|---|---|---|---|---|---|---|---|
| | | | | | | | literacy and the teach-back method | | | | | retained skills and knowledge. | | | |
| Keisling BL, Bishop EA, Roth JM. Integrating Family as a Discipline by Providing Parent Led Curricula: Impact on LEND Trainees' Leadership Competency Matern Child Health J May 2017;21(5):1185-1193 | 2017 | USA | Graduate students (psychology, speech-language pathology, audiology, nutrition, social work, nursing, and family) (N=127) | Children with neurodevelopm ental disabilities | 8-12 hours with the family | Part of LEND education (Leadership Education in Neurodevelopmental and related Disabilities), family members employed, students spending 8–12 h with the mentoring family, journaling those experiences, and participating in monthly interdisciplinary conversations which focus on a variety of current issues in disability. | Spending 8–12 h with the mentoring family, journaling those experiences, participating in monthly interdisciplinary conversations which focus on a variety of current issues . Examples of topics: The Financial Impact of a Child with a Disability; Cultural Aspects of Self-Determination; Long-Term Supports: Who Takes Over When the Parents are Gone? | Increased family-centred competence, including an awareness of multiple issues faced by families and a growing appreciation for a diverse cross-section of family structures. | Yes | Pretest-Posttest Design, mixed-method | MCH (Maternal and Child Health) Leadership Competencies (version 3.0) as a pre- and post-test measured by likert scale 1-5 + comments | Significantly higher across all family-centred leadership competency items at the completion of their traineeship. Positive comments. | Family-centred care, medical home model of primary care | n/a (LEND, program across several states in the USA funded by the Maternal and Child Bureau) | 1+2 |

| | | | | | | | | | | | | | | | |
|---|---|---|---|---|---|---|---|---|---|---|---|---|---|---|---|
| Khan A, Spector ND, Baird J, et. al. Patient safety after implementation of a coproduced family centered communication programme: multicenter before and after intervention study<br>Bmj Dec 5 2018;363():k4764 | 2018 | USA/Canada | Nurses (N=435), medical students (N=203), residents (N=586) | Inpatients | 1 hour faculty, 1 hour medical students, 2-3 hours residents, 15 minutes nurses (9 months implementation period) | Training conducted in-person, at resident orientation, staff meeting, individual or online. Slide deck, simulation/role-play exercises or video modules.Reinforcement and feedback provided individually. (Part of larger implementation strategy). | Family engagement strategies, nurse engagement strategies, roles on rounds, health literacy, I-PASS format (Illness severity, Patient summary, Action list, Situation awareness and contingency planning, Synthesis by receiver) | To improve and standardize communication on rounds by emphasizing health literacy, family engagement, and bidirectional communication | Yes | Multicentre Pretest-Posttest Design | Medical errors reviewed by blinded reviewers, research assistant live observations and surveys administered to parents 3 months before implementation and 3 months post intervention. | Rate of medical errors unchanged, harmful errors and adverse events decreased significantly, six of 25 components of family reported experience improved; none worsened, Family centred rounds occurred more frequently. Duration of rounds did not change significantly | Family-centred care, patient- and family-centred care | n/a | 3+4 |
| Khoo SA, Aswin W, Shen GQY, et al. Improving provider-patient communication skills among doctors and nurses in the children's emergency department<br>Asia Pacific Scholar 2020;5(3):28-41 | 2020 | Singapore | Doctors (N=185), nurses (N=110) | Children's Emergency Department | Workshop 3 hours. Web-based module 5 videos between 5-7 minutes. | A pre-workshop web-based, self-directed, learning module with videos on five different scenarios and at tutor-led workshop with sessions going through scenarios in the videos, real face-to-face session with simulated patients, and small group feedback session with content specialists | The commonest complaint themes: Perception of waiting time, handling of dissatisfied patients, information delivery and expressive quality, physician's attitude and lack of empathy/ inappropriate use of body language, physician's explanation of illness and treatment. Concept: 'I Hear You' (Introduce, identity, information gathering, patient's perspective, patient's language, agreement, closure). Scenarios: long waiting time, | Improve the communication skills, increase the level of confidence amongst emergency medicine personnel in dealing with communication issues and to reduce communications related negative patient feedback in the Children's ED | Yes | Mixed method | Feedback forms and focus group interviews + patients' satisfactory surveys | Significant (81.8%) reduction in communication-related negative feedback monthly, 95% of the participants felt that they were able to better frame their communications. Focus group four main themes: 1 Increased empowerment of staff; 2 Improved focus of communication with parents; 3 Reduced feeling of incompetence when dealing | Patient-centred care, blended learning | n/a | 1+2+**3**+4 |

| | | | | | | | | | | | | | | | |
|---|---|---|---|---|---|---|---|---|---|---|---|---|---|---|---|
| | | | | | | | lost full blood count sample, patient education, medication error, patient management and delivery of medications. | | | | | with difficult parents; 4 Increased understanding of main issues and parental needs. | | | |
| Kind T, Goldman E, Fratantoni K, et al. Learning to deliver care in a medical home: a qualitative analysis of residents' reflections on practice<br>Clin Pediatr (Phila) Jun 2014;53(7):658-65 | 2014 | USA | Senior paediatric residents (N=27) | Children with special healthcare needs | n/a | Voluntary online course, 4 modules, reflection questions after each module | Content of the 4 modules: (1) practicing in a medical home (2) understanding care plans and applying the "Always Event" (defined as the physician and family agreeing on next steps and specific responsibilities at the end of each clinical encounter) (3) understanding and identifying community resources for children with special healthcare needs (4) | Acquire the necessary knowledge, attitudes, and skills to deliver effective, patient-centred care | Yes | Qualitative | 4 reflective questions posted online on course web-site | Residents realised they needed to better understand families' financial, emotional, and social needs, and needed to increase the involvement of families in care planning. Lastly, they identified systems issues and practice changes they themselves could make to | Patient-centred care, medical home, patient- and family-centered care, family-centred care | n/a | 2 |

| | | | | | | | | | | | | | | | |
|---|---|---|---|---|---|---|---|---|---|---|---|---|---|---|---|
| | | | | | | | developing a care plan. | | | | | improve care delivery. | | | |

| | | | | | | | | | | | | | | | |
|---|---|---|---|---|---|---|---|---|---|---|---|---|---|---|---|
| King G, Tam C, Fay L, et. al. Evaluation of an occupational therapy mentorship program: effects on therapists' skills and family-centered behavior Phys Occup Ther Pediatr Aug 2011;31(3):245-62 | 2011 | Canada | Occupational therapists (N=25) (8 new and 17 experienced therapists) | Paediatric rehabilitation hospital (in-patient, out-patient, and community programs) | 11 months | New therapists: One-on-one mentor meetings biweekly 3 months, then monthly the next 3 months, and afterwards as needed. Experienced therapists: Encouraged to work with a mentor. For all: Peer mentorship meetings three times a month. Everyone should attend at least eight times and present at least one case study or topic of interest.. | One-on-one mentorship: observation, practice, knowledge acquisition, resource materials. Group intervention: Facilitated discussion of case studies. Both designed to create reflective practice. Guidelines for cases in group: occupational performance issues and interventions, best-practice evidence, client-centered considerations, community partnership, environmental considerations, communication strategies, relevant policy issues, reflection on learning | Facilitate the development of skills and behaviours known to be associated with service delivery expertise, including family-centred behaviour, critical thinking ability, listening/interactive communication skill, and clinical behaviour | No | Mixed Method Pretest-Posttest Design | Pre: Effective Listening and Interactive Communication Scale, Measure of Process of Care for Service Providers, Self-nomination Scale of Expertise in Paediatric Rehabilitation, and California Critical Thinking Disposition Inventory. 1-3 peers completed Peer Nomination Scale of Expertise & Multidimensional Peer Rating Scale. During intervention period: activity log. Post: all self-assessment questionnaires (except background form), peer assessment and focus groups. | Significant pre–post changes on 9 of the 12 measures examined, including measures of family-centred behaviour rated by therapists themselves, and also significant change in peer assessed cllinical skills. Focus groups revealed benefits of program, but did not experience it as affecting their practice. | Family-centred behaviour, developmental model | n/a | 1+2+3 |

| | | | | | | | | | | | | | | |
|---|---|---|---|---|---|---|---|---|---|---|---|---|---|---|
| Leaming-Van Zandt KJ, Zhu H, Banuelos RC, Lopez MA, Hsu DC. Impact of a Pediatric-Focused Communication Course on Patient/Caregiver-Perceived Physician Communication Skills in a Pediatric Emergency Department. Pediatr Emerg Care. 2021 Dec 1;37(12):e1173-e1180 | 2021 | USA | Paediatric Emergency Department Physicians (N=49) | Paediatric Emergency Department | 5.5 hours | "Breakthrough Communication" led by 2 practicing clinicians, facilitators guided learners through a series of short didactic presentations followed by small-group roleplay scenarios, session concluded with an integrative case where participants offered challenging clinical encounters from their practice setting, identified themes in communication challenges, and then selected one of these encounters to roleplay. | Based on the Academy of Communication in Healthcare model, communication skills to 3 segments of the clinical encounter (beginning the encounter, relationship-centered interviewing, and ending), active inclusion of the pediatric patient within clinical discussions, reflective listening and empathic skills, caregiver needs and expectations, recognizing complex family dynamics as triggers for shifts in communication technique, communication skills to build and enhance the pediatric "triadic" relationship | Improve physicians interpersonal and communication skills | No | Pretest-Posttest Design | Paediatric Physician Interpersonal Communication Skills Assessment [P-PICSA] by patient/caregiver | Post course scores were higher for all 13 items, with 3 items achieving statistical significance: (1) the doctor used words I could understand; (2) the way the doctor involved me in making decisions about my child's care in the ED; (3) how the doctor discussed next steps and/or follow-up plans for my child's care after we leave | Relationship-centred communication | n/a | 4 |

| | | | | | | | | | | | | | | |
|---|---|---|---|---|---|---|---|---|---|---|---|---|---|---|
| Lewis KD, Destino L, Everhart J, Subramony A, Dreyer B, Allair B, Anderson M, Baird J, Bismilla Z, Good B, Hepps J, Khan A, Kuzma N, Landrigan CP, Litterer K, Sectish TC, Spector ND, Yin HS, Yu CE, Calaman S, O'Toole JK. Patient and Family-Centered I-PASS SCORE Program: Resident and Advanced Care Provider Training Materials. MedEdPORTAL 2022;18():11267 | 2022 | USA | Residents (N=246) | General | 90 minutes +45 for self-study | Self-study training prior (PowerPoint video, written exercise, evaluation). In-person workshop or online: "The Patient and Family-Centered I-PASS (Illness severity, Patient summary, Action list, Situation awareness and contigency planning, Synthesis by receiver) Safer Communication on Rounds Every Time (SCORE) Program" (PowerPoint, discussion, role-play, written exercise, video, evaluation.) | Self-study: background information on patient- and family-centred rounds, history of patient- and family-centred I-PASS, how to incorporate it, importance of communication and health literacy. Workshop: discussion of health literacy exercise, practice through interactive exercises of the structured communication on rounds, roles and responsibilities of all team members on rounds, considerations for situations that may impact rounds communication, advanced communication techniques, considerations based on developmental age, limited English proficiency, discussion of sensitive topics, teaching on rounds, video example of good/bad round | Teaching residents and including important techniques for patient- and family-centred rounds that can promote general communication, the interprofessional care team, shared understanding, and, importantly, patient safety. | Yes | Cross-sectional | 2 questionaires (self-created). The first regarding attitudes about skills and knowledge within 5 objectives & the second was feedback on the training | Above 80% agreed or strongly agreed that the training provided knowledge and confidence in skills ability for all five stated objectives, had appropriate balance of didactic and interactive elements. 80% agreed or strongly agreed that it had an apprpriate pace and 75% thought it had the correct lenght to adress the content. | Patient- and family centred care, family-centred care, patient- and family-centred rounds, family-centred rounds, Kern's six steps for curriculum development, Kirckpatricks model | n/a | 1+2 |

| | | | | | | | | | | | | | | | |
|---|---|---|---|---|---|---|---|---|---|---|---|---|---|---|---|
| Mandak K, Light J, McNaughton D. The Effects of an Online Training on Preservice Speech-Language Pathologists' Use of Family-Centred Skills Am J Speech Lang Pathol Aug 4 2020;29(3):1489-1504 | 2020 | USA | Speech-language pathology master's students (N=17) | Children with complex communication needs | Average of 61 minutes | Online training. Four sections (introduction, LAFF strategy instruction, practice activities, and conclusion).The training was housed on Moodle (e-learning platform). | Relational skills strategy: LAFF= (a) Listen, empathize, and communicate respect; (b) Ask questions; (c) Focus on the issues; and (d) Find a first step. | To teach preservice speech-language pathologists how to actively listen and communicate effectively with parents of children with complex communication needs. | No | Randomised 2 × 3 experimental design (2 groups x 3 times. Group 1: training between time 1 & 2; Group 2: training between time 2 & 3) | Both groups were videotaped during interactions with simulated parents three times. Scoring rubric by two individual coders blinded, parent blinded viewed videos and assessed them, questionnaire for the participants post training. | Significant change in relational skills assessed by two independent coders, parent selected 14 of 15 post-test videos as more family-centred than pre-test and, 100% of the students would recommend the use of the strategy to others | Family-centred practice, strategy instruction model | n/a | 1+3 |
| McArdle GK, McDermott MR. From directive expert to non-directive partner: A study of facilitating change in the occupational self-perceptions of health visitors and school nurses. British Journal of Guidance & Counselling 1994;22(1):107-117 | 1994 | UK | Health visitors and school nurses (N=24, 12 training group and 12 in control group) | Children with special healthcare needs | 10 weeks ( 3 hours per week) | Course run by two qualified clinical psychologists. Training scenarios facilitated and illustrated behavioural principles with respect to child management. Videomaterial, role-play, task analysis, reading materials, homework tasks, group discussions. | "The Parent Advisor Scheme: Manual for Training Counsellors to Work with Families of Children with Special Needs" (Davis et al., 1987) ie the needs and characteristics of families, parent-proffessional relationships, methods of recording and observing carer-child interactions, difficulties encountered when attempting to operantly shape up children's behaviour, task analysis in order to understand the complexity of everyday activities. Homework tasks: ie observation of the use and consequenses of | Shift in perception from "expert" to "partner", change self-perceptions of counselling skills, professional effectiveness, communication skills, stress and support, locus of control and self-esteem. | No | Pretest-Posttest Design with control group | Three questionnaires pre training and one month after. (Training group also immediately after). 1) Self-created; 2) An adapted form of Levenson Locus of Control Scale; 3) Rosenberg unidimensional measure of self-esteem | Significant difference between training and no-training group on self-perceived counselling skills and self-perceived communication skills. Significant difference in 5 out of 8 variables for training group between pre and post training. | Partnership model | n/a | 2 |

| | | | | | | | | | | | | | | | |
|---|---|---|---|---|---|---|---|---|---|---|---|---|---|---|---|
| | | | | | | | empathy within conversations | | | | | | | | |

| | | | | | | | | | | | | | | |
|---|---|---|---|---|---|---|---|---|---|---|---|---|---|---|
| Meyer EC, Brodsky D, Hansen AR, Lamiani G, Sellers DE, Browning DM. An interdisciplinary, family-focused approach to relational learning in neonatal intensive care. J Perinatol. 2011 Mar;31(3):212-9. | 2011 | USA | Physicians, nurses, social workers, psychologists, chaplains and medical interpreters (N=74) | NICU (neonatal intensive care unit) | 6 hours | Program to Enhance Relational and Communication Skills–Neonatal Intensive Care Unit (PERCS-NICU). Workshop: Collaborative exercise, educational film, didactic presentation, a neonatal case scenario portrayed by professional actors interacting with voluntary staff, peers observed through live video feed, reflections and feedback. Closed with ideas they planned to incorporate into their own clinical practice | Collaborative exercise: communication strategies they had found helpful in their own clinical practice. Film: family members described experience with end-of-life care for their child. Didactic presentation: common communication challenges, approaches for conveying difficult news, ethical guidelines for withdrawal of life-sustaining therapy. A neonatal case scenario. Interdisciplinary teams volunteered to interact with the 'family' during each of three successive conversations, their peers observed the discussion through live video feed, feedback afterwards, shared ideas they would incorporate to their own practice. | To enhance relational and communication skills | Yes | Mixed Method Pretest-Posttest Design | Questionnaires baseline, post training and 12 Months follow-up, self created, used 5 point Likert scale + open-ended questions. | Improved preparation, communication skills, confidence, improved ability to establish relationships, reported reduced anxiety (16-100%). Qualitative themes included integrating new communication and relational abilities, honoring the family perspective, appreciating interdisciplinary collaboration, personal/human connection and valuing the learning. 93% applied skills learned, 74% transformed practice and 100% recommended the workshop | Relational learning, family-centred care | n/a | 1+2+3 |

| | | | | | | | | | | | | | | | |
|---|---|---|---|---|---|---|---|---|---|---|---|---|---|---|---|
| Montgomery L, Benzies K, Barnard C. Effects of an Educational Workshop on Pediatric Nurses' Attitudes and Beliefs About Family-Centred Bedside Rounds. J Pediatr Nurs. 2016 Mar-Apr;31(2):e73-82. | 2016 | Canada | Nurses (N=56) | Paediatric inpatients | 2 hours | Workshop: Video, reaction sharing, review of literature, probe questions, small group discussions, brainstorming, tip sheet | Video that showed the importance of relational practice and value of patient- and family-centered care, a review of the literature to provide evidence of the value of Family-centred Bedside Rounds, video demonstrating an example, and literature that described skills nurses can use during Family-centred Bedside Rounds. | To influence nurses' intentions to actively participate in bedside rounds using a multifaceted approach addressing the predictors (i.e.attitudes,subjective norms, and perceived behavioral control) of intention to practice a desired family-centred bedside round behavior | No | Pretest-Posttest Design | Nurses Attitudes and Behaviours about Rounds (NABAR) questionnaire (created and validated for the study), administered before workshop and six weeks after. | Significant increases between pre-test and post-test scores on nurses' intentions, subjective norms, perceived behavioural control, and in practicing open communication, and providing education for families. | Theory of planned behaviour, family-centred care, patient- and family-centred care | n/a | 2+3 |

| | | | | | | | | | | | | | | | |
|---|---|---|---|---|---|---|---|---|---|---|---|---|---|---|---|
| Mutambo C, Shumba K, Hlongwana KW. Post-training and mentorship experiences of KidzAlive-trained healthcare workers at primary healthcare facilities in KwaZulu-Natal, South Africa<br>Afr J Prim Health Care Fam Med Jun 29 2020;12(1):e1-e11 | 2020 | South Africa | Nurses and HIV counsellors, (N=1411) | Children with HIV (human immunodeficiency virus) | 5 days + time spent with mentor | KidzAlive training: Classroom training covering educational content. With mentor: Healthcareworker booked 8 child-caregiver pairs, 3 cared for by mentor, healthcare worker observed, 5 by healthcare worker, supervision of mentor. Mentor used checklist to certify. | Child rights, play therapy techniques, techniques for communicating with children, support mechanisms for primary caregivers, child-friendly spaces, stages of childhood development and dealing with children in distress. | Improve the quality of HIV care among children | Yes | Qualitative | 6 months after training individual structured interviews | (1) increased healthcare worker knowledge, skills and confidence to provide child-friendly HIV services to children (2) increased involvement of HIV+ children in own healthcare journey (3) the involvement of primary caregivers in children's healthcare journey (4) improved health outcomes for HIV+ children 5) transformation of the primary healthcare environment towards being more child-friendly | Child-centred care, patient-centred care, social learning theory, cognitive developmental theory, sociocultural theory, the Play Therapy Theory | Endorsed by the South African National Department of Health | 1+2+3 |

| | | | | | | | | | | | | | | | |
|---|---|---|---|---|---|---|---|---|---|---|---|---|---|---|---|
| Nehal US, Kanahara S, Tanabe M et al. Pediatric Refugee Health Care Delivery in the Community Setting: An Educational Workshop for Multidisciplinary Family-Centered Care During ResettlementMedEdPORTAL Nov 3 2020;16():10988 | 2020 | USA | 3 hours workshop: Clinicians, nurse practitioners, paediatric residents, medical students, undergraduate students, (N=17). 1 hour workshop: clinicians, public health professionals and policy makers, (N=more than 30). | Refugees | 3 hours/1 hour shortened version | Workshop: presentations, discussion, clinical case scenarios, small group case discussions followed by large-group report-back, personal goal-setting | Experiences and sources of trauma that refugees might experience prior to arrival, common medical conditions faced by refugees (discussion of determining vaccination status, overcoming cost barriers to care, importance of shared decision-making), providing trauma-informed care when addressing the needs of at-risk children (SAFE model), policy, advocacy, and outreach, | 1) Identify key aspects of physical, behavioral, and social health that affect pediatric refugee patients. 2) Decide what diseases to screen for and what treatments to give to refugee patients prior to arrival. 3) Plan whole-person care for pediatric refugee patients that is trauma informed and culturally sensitive. 4) Improve effectiveness as an advocate for funding and resources needed to deliver care in their own setting. | No | Pretest-Posttest Design | Four self-assessment questions pre- and post-study + evaluation forms post study. | 3 hours workshop: Comfort asking about social and behavioural health increased significantly, the rest insignificant. 1 hour workshop: Significant better understanding of prearrival experiences after workshop, the rest insignificant. Both 1 hour and 3 hours workshop learners rated workshop between 4-5 on a five point likert scale (nearly excellent). (Small sample size 5-23). | Bloom's taxonomy, family-centred care, shared decision making, SAFE model | n/a | 1+2 |

| | | | | | | | | | | | | | | | |
|---|---|---|---|---|---|---|---|---|---|---|---|---|---|---|---|
| Newes-Adeyi G, Helitzer DL, Roter D, Caulfield LE. Improving client-provider communication: evaluation of a training program for women, infants and children (WIC) professionals in New York state. Patient Educ Couns Nov 2004;55(2):210-7 | 2004 | USA | Nutrionists (WIC providers, WIC = Women, Infants and Children), (N=35) | Children under 5 years at nutritional risk | 1 day | Lecture, case studies, discussion, small group work, and role-plays. The training focused on a set of key communication behaviors. | Growth monitoring charts, nutrition education messages, appropriate ways to impart these messages, patient-centered approach, communication behaviors to help the provider elicit the mother's perspective on her child's health, and negotiate a follow-up plan with her. Seven-step approach: taking history, eliciting client's perspective, responding to perspective, educating, negotiating plan, discussing problems with carrying out plan, solving identified problems | Enhance eliciting the client's perspective on the issues discussed, negotiating follow-up strategies, discussing problems and potential solutions to these, maintaining a balance between provider and client talk, and using open-ended questions. | Yes | Pretest-Posttest Design | Audiotaped mock counselling sessions with a simulated client (trained actress) before and after, analysed with Roter Interaction Analysis System + demographic data questionnaire and questionnaire about satisfaction with the interaction (modification of validated scale). | Significantly asked more open-ended questions, provider-to-client-talk ratio decreased, eliciting client perspective increased and provider competence-related satisfacion increased. | Patient-centred approach | n/a | 2+3 |

| | | | | | | | | | | | | | | |
|---|---|---|---|---|---|---|---|---|---|---|---|---|---|---|
| Pawłowska M, Del Rossi L, Kientz M, McGinnis P, Padden-Denmead M. Immersing students in family-centred interprofessional collaborative practice J Interprof Care Jan-Feb 2020;34(1):50-58 | 2020 | USA | Students (N=111) (occupational therapy (N=29), physical therapy (N=22), nursing (N=28), speech-language pathology (N=32) | Healthy infants/toddlers 4-28 months | A half day | "Baby Day": Families recruited from community.Half hour team meeting (2-3 students). Shared behaviours they would like to observe and negotiated a plan for team–family interaction. Implemented the plan during a 1-h play-based interaction with a child and parent/caregiver and interview of accompanying parent/caregiver. Afterwards small group debriefing followed by large group debriefing. | Students interacted with an infant/todler and one or two parents/caregivers aiming to develop a trusting relationship with parents, families and other healthcare team members, respect the cultures, values, roles and responsibilities, and experience of other professions, listen actively and encourage ideas & opinions of family and other team members, communicate with baby/family and other team members in a responsive and responsible manner that supports a team approach. Tasks: set 2–3 personal learning objectives for themselves, list age appropriate skills they wanted to observe, draft questions they wanted to ask the parent, select activities and play/toy ideas their assigned child might enjoy. | Support the development of team and child/family interaction skills and the understanding of the value of a team approach in early intervention | Yes | Mixed method | Parent survey and student evaluation forms post training. During training: The Interprofessional Collaborator Assessment Rubric by four independent observers for eight randomly selected student teams + self-ratings by these students post intervention. In addition four reflective questions for all students within a week. | 96.6% of students rated the overall Baby Day experience as good or excellent. Quantitative rating scale data indicated consistency between family, student and independent observer ratings of family-centred care. Qualitative data suggested that students gained a better understanding of ways in which an interprofessional team can provide effective family-centred care For the majority of the ICAR dimensions, students rated themselves as competent, but did not rate themselves as displaying mastery of any skil. | Patient-as-partner, patient/family-centred care, family-centred care | n/a | 1+2 |

| | | | | | | | | | | | | | | |
|---|---|---|---|---|---|---|---|---|---|---|---|---|---|---|
| Roter DL, Larson S, Shinitzky H, et al. Use of an innovative video feedback technique to enhance communication skills training Medical Education 2004;38(2):145-157 | 2004 | USA | Paediatric residents (N=28) | Inpatients (Children hospitalized after a crisis with asthma, diabetes or sickle cell disease.) | 4 hours spread on 4 weeks | Simulated interview videotaped. One-hour didactic and role-playing practice session. One hour reviewing the coded videotape within an interactive CD-ROM platform focusing on areas of communication related to the prior week's didactic and roleplaying session. A second simulated interview was conducted in the fourth week. | Simulated interview: discussing discharge planning and follow-up care with parent of a child who had been hospitalized after a crisis with asthma, diabetes or sickle cell disease. Didactic: the Three Function Model of Medical Interviewing. Focus on 4 skills: listening more ⁄ talking less, data gathering techniques (open-ended questions), responding to the parent's emotions, building an active partnership for problem. Reviewing video with faculty preceptor receiving structured feedback on the 4 skills. | Teaching and learning of communication skills | No | Pretest-Posttest Design | Self-assessment checklist + coding of videotapes with RIAS (Roter Interaction Analysis System) software by two independent coders blinded to pre - post status | Significant reduced verbal dominance, increased use of open-ended questions, increased use of empathy, and increased partnership building and problem solving for therapeutic regimen adherence. Female residents demonstrated greater communication change than males. Residents found the feedback session to be helpful. 86% of the residents reported that the feedback session was productive in improving their clinical skills | Three Function Model of Medical Interviewing, partnership | n/a | 1+3 |

| | | | | | | | | | | | | | | |
|---|---|---|---|---|---|---|---|---|---|---|---|---|---|---|
| Su H, Llewellyn G, Yi Y, Gao Y, Liu, J. The feasibility of family-centred early intervention for children with disabilities in mainland China: Practitioners' perceptions Child: care, health and development 2021;47(6):869-876 | 2021 | China | Early intervention practitioners (speech therapist, teacher, occupational therapist) (N=37) + officers from the provincial disabled persons' federation (N=2) | Children with disabilities | 1 hour | Workshop: Presentation about family centred practices. | Presentation about family centred practices, including a theoretical introduction and description of it in Australia. The concept of a transdisciplinary key worker model (Early Childhood Intervention Australia, 2018) was introduced. | Presenting family-centred practices | No | Qualitative | Focus groups: Two open-ended questions: What kinds of services do your agencies provide to parents and families? What do you think of implementing family-centred practice in China as described in the presentation? | (a) family-centred early intervention is possible, (b) traditional concepts are not friendly towards family-centred practices (c) parents do not collaborate (d) financing and personal resources are not sufficient to implement it. Chinese practitioners agreed with the philosophies; however, there was concern that widespread implementation may meet conceptual and practical challenges | Family-centred practice, transdisciplinary key worker model | n/a | 1 |

| | | | | | | | | | | | | | | | |
|---|---|---|---|---|---|---|---|---|---|---|---|---|---|---|---|
| Taff H, Gilkerson L, Osta A, Seo-Lee A, Schwartz A, Chunara Z, McGinn L, Pillai N, Barnes MM. Strengthening Parent - Physician Communication: A Mixed Methods Study on Attuned Communication Training for Pediatric Residents.Teach Learn Med Aug 2022;():1-12 | 2022 | USA | Residents (N=23) | Paediatric primary care clinic | 6 months (Intro: 3 hours, Month 1+2: 3x 30 min or 2x 60 min, Month 3: 60 min, Month 4,5,6: 3x 30 or 2 x 60 minutes) | FAN (Facilitating Attuned iNteractions) curriculum: Initial 3 hour workshop in groups of 5-6 with didactics, videos, role play, small group discussions. Month 1 + 2: Individual mentor reflection session x 3 or group mentor sessions x 2 Month 3: Booster training. Month 4,5,6: Individual mentor reflection sessions x 3 or group mentor reflection sessions x 2 | The FAN model/FAN communication tool: 5 areas for communication, correlating a mental/emotional state with an associated communication strategy: Calming/Mindful Self-Regulation, Feeling/Empathic Inquiry, thinking/Collaborative Exploration, Doing/Capacity Building, Reflecting/Integration. Key element: identifying mismatches and re-attuning communication. Framework for the clinical encounter, includes pre-contact self-regulation and a defined beginning, middle, and end of the encounter that promotes collaboration throughout the visit | Developing the patient-physician relationship, operationalizing attunement by teaching physicians how to read the parent's cues and match their communication approach to the parent's demonstrated need. | No | Mixed-method | Residents: Jefferson Scale of Physician Empathy, the Kentucky Inventory of Mindfulness Skills (1,3,7,9,12 months), one semi-structured interview at end of study. Parents: Jefferson Scale of Patient Perceptions of Physician Empathy, Consultation and Relational Empathy (CARE) Patient Feedback Measure (1,3,7,9,12 months). Mentors: Mentor Review Forms, one semi-structured interview at end of study. | Relational empathy (CARE) scores increased significantly, the other 3 instruments insignificant. Interviews: improved attunement with families, improved clinical skills due to increased collaboration with families, increased efficiency in clinical encounters. Mentors reported use of communication tool with high frequency up to 6 months after initial training. | patient-centred communication, patient- and family-centred communication,Facilitating Attuned Interactions model, patient-centred care | n/a | 2+3+4 |

| | | | | | | | | | | | | | | | |
|---|---|---|---|---|---|---|---|---|---|---|---|---|---|---|---|
| Toivonen M, Lehtonen L, Ahlqvist-Björkroth S, Axelin A. Key factors supporting implementation of a training program for neonatal family- centred care - a qualitative study. BMC Health Serv Res. 2019 Jun 19;19(1):394. | 2019 | Finland | The entire staff (nurses, neonatologists, paediatricians) (N= 8 neonatal units) | NICU (neonatal intensive care unit) | 1.5 year | The Close Collaboration with Parents™ Training Program: 2-4 nurses trained as mentors by trainer team (a psychologist, neonatologist, and nurse). 4 phases (1. Observation of infant behavior. 2. Joint observation. 3. Individual story of the family. 4. Family centered transition to home.) Each phase started with one week theoretical teaching (lectures, demonstrations, small group practice) for entire staff. So mentoring of mentor nurses, and mentor nurses mentoring the rest of the staff, supported by training manual. Lastly, group discussions and reflection. | 4 phases: 1) observation of infant behavior, identify indvidual features and preferences 2) actively listen to parents´perceptions through joint observation, collaborative planning of care, partnership 3) individual story of family/infant, empathy, individualized plan for parental participation, partnership 4) collaborative planning of transition to home, shared decision-making, partnership. | Facilitate the staff to work with the parents and promote family-centred care. Change the care culture so that parents are accepted as partners in infant care. | Yes | Qualitative | 16 focus group interview (managers/doctors and nurses, separate) and two individual semi-structured interviews | Elements that appear to have been positive to implementation of the training program were flexible and motivated mentor nurses who supported the change, team commitment of the whole unit and support from the leadership. Barriers to implementation included inadequate preparation for the change and non-supportive unit design. | Family-centred care, experiential learning theory, i-PARIHS framework | n/a | 1+2+3 |

| | | | | | | | | | | | | | | |
|---|---|---|---|---|---|---|---|---|---|---|---|---|---|---|
| Toivonen M, Lehtonen L, Löyttyniemi E, Ahlqvist-Björkroth S, Axelin A. Close Collaboration with Parents intervention improves family-centred care in different neonatal unit contexts: a pre-post study. Pediatr Res. 2020 Sep;88(3):421-428. | 2020 | Finland | The entire staff (nurses, neonatologists, paediatricians) (N= 8 neonatal units) | NICU (neonatal intensive care unit) | 1.5 year | The Close Collaboration with Parents™ training program: See description in the article above. | The staff learn to observe infant behavior to identify each infant's individual needs and features; to listen to parents' perceptions about their infant and to give them psychological space to create a shared care plan; to understand the individual story of becoming the parents; and to integrate parents in decision making, especially during the transition from hospital to home. | Developed to support parenting and parent–infant attachment by increasing collaboration between parents and staff and by improving the quality of family-centred care. | Yes | Mixed-method Pretest-Posttest Design | Bliss Baby Charter audit tool (not validated) by nurses, unit managers and parents. Semi-structured focus group interviews (nurses, unit managers, and parents separate) | The quality of family-centred care, assessed by staff and parents, increased significantly after the intervention. The intervention was able to help staff define and apply elements of family-centered care, such as shared decision making and collaboration between parents and staff. In interviews, staff described that they learned to support and trust the parents' ability to take care of their infant. | Family-centred care | n/a | 2+3+4 |
| Walter L, Robb M. Promoting Discharge Readiness Through Staff Education: A Family-Centred Approach J Nurses Prof Dev May/Jun 2019;35(3):132-136 | 2019 | USA | Nurses ( N=180) | NICU (neonatal intensive care unit) | 30 minutes lecture + 4 weeks mentoring | 30 minutes lecture: PowerPoint about family-centred care (presentation, case examples and discussion). Also online option. 4 weeks mentoring in the unit by project manager afterwards. Posters in the unit, infographic card, small charm | Addressed the central tenets of family-centred care, methods of integrating it into daily practice, barriers and means of overcoming barriers, the impact of family-centred care on parental perception of discharge readiness | Increase nurses knowledge of family-centred care practices and to empower them to integrate the principles into their daily patient care practices. | Yes | Pretest-Posttest Design | Self-designed knowledge test. Scale 0-10. Content validated by experts. Completed baseline, post lecture, and 4 weeks after lecture. | Significant change (one-tailed paired t-test) 8.15 (SD 1.23), n=159 to 9.97 (SD 0.21), n=61. After 4 weeks 9.62(SD 0.82), n=65. | Family-centred care | n/a | 2 |

| | | | | | | | | | | | | | | |
|---|---|---|---|---|---|---|---|---|---|---|---|---|---|---|
| Weis J, Zoffmann V, Egerod I. Improved nurse-parent communication in neonatal intensive care unit: evaluation and adjustment of an implementation strategy J Clin Nurs Dec 2014;23(23-24):3478-89 | 2014 | Denmark | Nurses (N=45) | NICU (neonatal intensive care unit) | 1 day + supervision during working hours 3-21 months | 1-day workshop (see "Educational content"). Supervision and feedback sessions during working hours with voice-recorded nurse-parent dialogues, case report forms and individual training schedules. | One-day workshop: Familiarised nurses with the main components and theoretical underpinnings of guided family-centred care, presenting theory related to nurse–parent communication, family-centred care, guided self-determination and person-centred communication | Implementation of guided family-centred care and ensure adherence. | No | Mixed-method | Written knowledgetest after wokshop, interviews with trainees, final written certification test (12 multiple-choice questions on theory related to guided family-centred care, six open-ended questions and two unfinished sentences). | All nursed passed the knowledge test post workshop. In interviews nurses acknowledged the importance of guided family-centred care, usefulness of reflection sheets and that voice recordings facilitated learning. 25 of 45 nurses passed the certification test. Not passing the test was mainly because of educational or maternity leave. | Guided family-centred care, family-centred care, guided self-determination, person-centred communication, empowerment | n/a | 2 |

| | | | | | | | | | | | | | | | |
|---|---|---|---|---|---|---|---|---|---|---|---|---|---|---|---|
| Weiss S, Goldlust E, Vaucher YE. Improving parent satisfaction: an intervention to increase neonatal parent-provider communication J Perinatol Jun 2010;30(6):425-30 | 2010 | USA | Principal medical providers (N=57)( 9 attending physicians, 7 NICU fellows, 37 paediatric residents and 4 neonatal nurse practitioners), nursing staff (N=?), NICU support staff (N=?), nursing leaders (N=?) | NICU (neonatal intensive care unit) | 5 minutes: NICU support staff and nursing leaders. 20 minutes: nursing staff. 30 minutes: principal medical providers | Principal medical providers: Slideshow about a communication plan and important principles of communication. Nursing staff: Overview of the pre-intervention survey results, and the introduction of the principal medical provider education module, contact cards and poster. NICU support staff and nursing leaders: 5 minutes presentation. (In addition, posters and contact cards in the unit). | Principal Medical Providers: Introduced a communication plan, establish a thread of family communication from an infant's admission to discharge, recommended they met with families on a daily basis initially, and at least weekly once the patients became stable, emphasized important principles of communication, including use of interpreters, contact frequency and essential elements of NICU family meetings, introduction to contact cards. Nursing staff: Overview of the pre-intervention survey results, and the introduction of the Principal Medical Provider module, contact cards and poster. NICU support staff and nursing leaders: Presentation 5 minutes | Increase principal medical provider availability and communication frequency | Yes | Pretest-Posttest Design | Pre- and post survey completed by parents. Based on 6 content-validated Likert scale questions, written by Press Ganey and the Picker Institute, addressing availability, understanding, reciprocity and empathy. Revised by research team, which also added 6 open-ended questions about quantity of communication. | Significant more subjects in the post-intervention cohort were satisfied with provider communication than in the pre-intervention cohort (95% vs 74%; P<0.01). Parents who reported talking with a provider in the previous 7 days were more satisfied than parents who did not (P<0.001). After the intervention, fewer families (36% versus 65%) reported a desire for more frequent provider contact (P<0.01). | Family-centred care | n/a | 4 |

| | | | | | | | | | | | | | | | |
|---|---|---|---|---|---|---|---|---|---|---|---|---|---|---|---|
| Wissow L, Gadomski A, Roter D, Larson S, Lewis B, Brown J. Aspects of mental health communication skills training that predict parent and child outcomes in pediatric primary care. Patient Educ Couns. 2011 Feb;82(2):226-32. | 2011 | USA | Physicians & nurse practitioners (N=31) | Children (5-16 years) with emotional and behavioural problems in primary care | 4 hours (three sessions spaced about three weeks apart) | 60 minutes small group discussion, on-site, led by a child psychiatrist. Used a slide set containing background material, written and video examples of skills, and programmed pauses for interaction. Immediately after each discussion, practiced skills in a 10-minute standardised patient visit.Videos of these visits were returned to providers for guided self-assessment. | Skills for visit time management, pointing out parallels between standard pediatric diagnostic and treatment principles and mental health care, skills for working with parent and child hopelessness and anger, building a patient-provider relationship including mutual agenda setting and joint formulation of problems, influencing behavior involved techniques for developing and presenting advice and managing resistance | 1) Improve children's emotional and behavioral problems 2) Enable providers to increase patient expectations for positive outcomes, reach agreement on the nature of problems and desired treatment, and influence behavior change 3) Improve providers' expectations surrounding mental health care | No | Randomised controlled trial | Strengths and Difficulties Questionnaire (by youths 11-16 years, parents & teachers). 10 minute video recorded interview with a standardised parent and teenager coded by blinded coders, prior intervention and 4 weeks after. | Trained clinicians used significant more agenda setting, time, and anger management skills than controls and showed increased patient centeredness toward parents, but not adolescents. Increased patient-centeredness toward parents predicted improvement in child/youth symptoms and functioning (rated by parents), and improvement in youth-rated symptoms, significantly. | Patient-centred care/patient-centredness, Miller's competency pyramid | n/a | 3+4 |

| | | | | | | | | | | | | | | | |
|---|---|---|---|---|---|---|---|---|---|---|---|---|---|---|---|
| Zengin Akkus P, Ilter Bahadur E, Coskun A, Koken G, Karahan S, Ozmert EN. Family-centred service: Perspectives of paediatric residents from a non-Western country. Child Care Health Dev. 2020 May;46(3):275-282. | 2020 | Turkey | Paediatric residents (N=110) | General (paediatric wards, clinics, and emergency rooms) | 2 hours | Workshop: Lecture.Presentation of examples, and discussion. After the session, guidance about online resources, articles and handouts. | First part of the session (didactic education) focused on the definition, core principles, history, and outcomes of family-centred services + important remarks from the literature review and best practice models. After the didactic education,examples from literature and daily life and strategies to handle these cases in a more family-centred way discussed. Then ideas of participants about family-centred service and their suggestions on implementing it. | Increase the awareness of family-centred services | No | Pretest-Posttest Design | Prior to workshop & 6 months after: Measure of processes of care for service providers (MPOC-SP) and questionnaire about perspectives towards family-centred services designed to study. | Statistically significant increases in MPOC-SP scores suggesting improvements in self-reportedfamily-centred practices. Percentage of participants describing themselves as knowledgeable and competent increased. | Family-centred care, family-centred practices, patient- and family-centred care, family-centred service, family-centred approach | n/a | 2+3 |
